# Effectiveness of Digital Interventions for Reducing Behavioral Risks of Cardiovascular Disease in Nonclinical Adult Populations: Systematic Review of Reviews

**DOI:** 10.2196/19688

**Published:** 2021-05-14

**Authors:** Natalie Gold, Amy Yau, Benjamin Rigby, Chris Dyke, Elizabeth Alice Remfry, Tim Chadborn

**Affiliations:** 1 Public Health England London United Kingdom; 2 Centre for Philosophy of Natural and Social Science London School of Economics and Political Science London United Kingdom; 3 Centre for Diet and Activity Research MRC Epidemiology Unit University of Cambridge Cambridge United Kingdom; 4 Department of Sociology University of Durham Durham United Kingdom; 5 Department of Social Science Institute of Education University College London London United Kingdom; 6 Global Health Section Department of Public Health University of Copenhagen Copenhagen Denmark

**Keywords:** alcohol, behavior change, cardiovascular disease, diet, digital interventions, digital medicine, internet interventions, mHealth, mobile interventions, physical activity, smoking, tobacco, mobile phone

## Abstract

**Background:**

Digital health interventions are increasingly being used as a supplement or replacement for face-to-face services as a part of predictive prevention. They may be offered to those who are at high risk of cardiovascular disease and need to improve their diet, increase physical activity, stop smoking, or reduce alcohol consumption. Despite the popularity of these interventions, there is no overall summary and comparison of the effectiveness of different modes of delivery of a digital intervention to inform policy.

**Objective:**

This review aims to summarize the effectiveness of digital interventions in improving behavioral and health outcomes related to physical activity, smoking, alcohol consumption, or diet in nonclinical adult populations and to identify the effectiveness of different modes of delivery of digital interventions.

**Methods:**

We reviewed articles published in the English language between January 1, 2009, and February 25, 2019, that presented a systematic review with a narrative synthesis or meta-analysis of any study design examining digital intervention effectiveness; data related to adults (≥18 years) in high-income countries; and data on behavioral or health outcomes related to diet, physical activity, smoking, or alcohol, alone or in any combination. Any time frame or comparator was considered eligible. We searched MEDLINE, Embase, PsycINFO, Cochrane Reviews, and gray literature. The AMSTAR-2 tool was used to assess review confidence ratings.

**Results:**

We found 92 reviews from the academic literature (47 with meta-analyses) and 2 gray literature items (1 with a meta-analysis). Digital interventions were typically more effective than no intervention, but the effect sizes were small. Evidence on the effectiveness of digital interventions compared with face-to-face interventions was mixed. Most trials reported that intent-to-treat analysis and attrition rates were often high. Studies with long follow-up periods were scarce. However, we found that digital interventions may be effective for up to 6 months after the end of the intervention but that the effects dissipated by 12 months. There were small positive effects of digital interventions on smoking cessation and alcohol reduction; possible effectiveness in combined diet and physical activity interventions; no effectiveness for interventions targeting physical activity alone, except for when interventions were delivered by mobile phone, which had medium-sized effects; and no effectiveness observed for interventions targeting diet alone. Mobile interventions were particularly effective. Internet-based interventions were generally effective.

**Conclusions:**

Digital interventions have small positive effects on smoking, alcohol consumption, and in interventions that target a combination of diet and physical activity. Small effects may have been due to the low efficacy of treatment or due to nonadherence. In addition, our ability to make inferences from the literature we reviewed was limited as those interventions were heterogeneous, many reviews had critically low AMSTAR-2 ratings, analysis was typically intent-to-treat, and follow-up times were relatively short.

**Trial Registration:**

PROSPERO International Prospective Register of Systematic Reviews CRD42019126074; https://www.crd.york.ac.uk/prospero/display_record.php?RecordID=126074.

## Introduction

### Background

The National Health Service (NHS) Long Term Plan sets out the UK government’s vision for preventing health problems and supporting the self-management of conditions [1]. A major target is cardiovascular disease (CVD), which causes 28% of all deaths in the United Kingdom and is the largest cause of premature death in deprived areas [2,3]. England’s primary large-scale intervention for CVD prevention is the NHS Health Check program [1,2], which was introduced in 2009 [4]. It is one of the largest public health prevention programs in the world, with over 6 million people in England having a check between 2013 and 2018 [5]. The NHS Health Check is a CVD risk assessment, which should be offered every 5 years to all adults aged between 40 and 74 years with no pre-existing vascular condition. As a result, people with previously undiagnosed conditions can be put on a clinical pathway and those who are at risk of developing a condition can be offered lifestyle support and advice to manage their risk. In particular, cardiovascular risk can be reduced by modifying 4 types of behavior: diet [6], physical activity [7], smoking [8], and alcohol consumption [9].

A key pillar of the Long Term Plan is *predictive prevention*—the use of technology and digital tools to identify health risks, make early diagnoses, and support positive health behaviors of those most at need through targeted treatments [3]. Predictive prevention, including the NHS Health Check, involves risk communication and behavior change. Evidence shows that risk communication alone does not lead to behavior change [10-13]. Therefore, we need to support behavior change. Digital tools are an increasingly important part of that landscape, and digital behavior change interventions may be offered to people after their NHS Health Check to manage their risk by helping them modify their diet, physical activity, smoking, or alcohol consumption.

Digital tools may be used either to supplement face-to-face services or to replace them. Replacement is particularly germane, since there is anecdotal evidence that face-to-face services are increasingly being defunded. In addition, services may need to shift from face-to-face to digital in response to the COVID-19 pandemic, which occurred after we had completed the review. Providers may hope that digital tools will offer a low-cost solution, with the potential to reach more people than traditional face-to-face services; however, the research base needs to be evaluated to see if there is sufficient evidence [14].

### Aims and Objectives

The first step is to establish whether digital interventions are effective. We also need to know which modes of delivery are most effective in order to allocate resources to develop the most promising digital tools or to know where research is needed, if the evidence base is lacking. In this systematic review of reviews, we aim to summarize the evidence on the effectiveness of digital interventions in improving dietary, physical activity, smoking, and alcohol consumption behaviors in nonpatient adult populations in high-income countries.

## Methods

### Overview

The study protocol was registered with PROSPERO (registration number: CRD42019126074). All deviations from the protocol are explained in the Methods section. We followed the PRISMA (Preferred Reporting Items for Systematic Reviews and Meta-Analyses) guidelines for reporting (see Multimedia Appendix 1 for the checklist) [15].

### Data Sources

Relevant reviews were obtained through an internet-based search and a manual search. First, 4 internet-based databases (MEDLINE, Embase, PsycINFO, and Cochrane Reviews) were searched for peer-reviewed review articles published in English between January 1, 2009, and February 25, 2019. We limited our search dates, only starting in January 2009, to make our study manageable and also because we expected reviews published in the last decade to capture earlier papers. Publications were restricted to English due to the absence of translation expertise. Gray literature searches were conducted in OpenGrey, ProQuest Dissertations and Theses, Google, and targeted websites (see Multimedia Appendix 2 for search terms and the gray literature search strategy). More articles were identified by manual searches of the reference lists from excluded reviews of reviews. We did not search in study registries, as we were looking for systematic reviews. We did not conduct full hand searches or consult experts to ascertain the literature for pragmatic and logistical reasons.

### Review Selection

The reviews were screened using a three-stage process. A total of 2 reviewers (NG and AY) examined titles and discarded reviews that did not meet the inclusion criteria (Textbox 1). Each reviewer then independently screened the abstracts of 10% of the remaining reviews to identify studies that potentially met the inclusion criteria. Interreviewer agreement on inclusion was also assessed. Reviewers disagreed on 11 of 41 decisions. All disagreements were resolved through discussion. AY screened all the remaining abstracts. The relevant review articles were then obtained in full and screened independently for eligibility by NG and AY. Any disagreement over eligibility was resolved through discussion with a third reviewer (TC or BR).

**Inclusion and exclusion criteria.**

Inclusion criteria

1. Study type: systematic reviews (whose reporting of the evidence could be either by narrative synthesis or by meta-analysis) that reported on the effectiveness of digital interventions in changing health-related behavior and/or health outcomes. We did not restrict by study design of the included studies within the systematic reviews.
2. Population: this included adult nonclinical populations. We aimed to assess the effectiveness of digital interventions in relation to CVD prevention relevant to the NHS Health Check program, which is offered to adults aged between 40 and 74 years (England, United Kingdom). Where populations were mixed, we included the review if the population of interest could be isolated. Where impossible to isolate the population of interest, reviews were included if ≥50% of the studies were of relevant populations.
3. Intervention: we included digital interventions targeting behaviors related to diet, physical activity, smoking, and/or alcohol consumption. Digital interventions include interventions delivered over the internet (web-based or websites), mobile telephone interventions (including texts and mobile apps), social media, computer-delivered interventions, and wearable technology. Interventions incorporating both digital and face-to-face components were also included.
4. Comparator: there were no restrictions. We extracted information about the comparators where available, allowing us to review effectiveness compared with both nondigital interventions and nonintervention controls.
5. Outcome: this included behavioral or health outcomes related to diet, physical activity, alcohol consumption, and smoking. Reviews that considered these areas of behavior, either individually or in combination, were included.
6. Time frame for follow-up: any time frame.

Exclusion criteria

1. Study type: reviews of reviews, conference abstracts, protocols, opinion pieces, and commentaries. We excluded reviews of reviews because we expected that most of the reviews gathered in a review of reviews would already be included in our study. Therefore, including reviews of reviews would have led to double counting of some information.
2. Population: reviews that only considered any of the following in ≥50% of included studies: children and adolescents, students, adults aged <40 years, pregnant women, management of existing CVD or other health conditions, and low- to middle-income countries. These criteria were selected to protect the ecological validity of this review, as relevant to the NHS Health Check.
3. Intervention: reviews of nondigital interventions. We did not consider television, radio, or telephone calls to be digital, as they are not often used in digital interventions for public health.
4. Comparator: no exclusion criteria.
5. Outcome: feasibility, acceptability, participation, and engagement only.
6. Superseded: this included reviews updated by subsequent reviews that included all the same studies as the original.

### Study Quality Assessment

Review confidence was critically appraised independently by 2 reviewers (NG and AY) for a 10% subsample of the included publications, using the AMSTAR-2 tool [16]. The findings were discussed to check for consistency. The remaining articles were divided and assessed by NG or AY. Any uncertainty was resolved through discussion with a third reviewer (BR).

### Data Extraction

Data were extracted using a standardized form (Multimedia Appendix 3). We extracted data on the following predefined review components: objective, population, inclusion and exclusion criteria, search date, included studies (number, type, and countries), follow-up, method of synthesis, results and findings, and comparator. A total of 2 reviewers (NG and AY) extracted the data independently for 10% of the publications. Discrepancies were resolved through discussion. The remaining publications were divided among both reviewers. During data extraction, we also noted information about the control condition and any information on a comparison of effectiveness of no-intervention versus active controls. Data on adherence and attrition were also recorded, where available. The data extraction form has been presented in Multimedia Appendix 3.

### Analysis

We conducted a systematic narrative synthesis using extracted data from included articles. No statistical analyses were conducted and meta-analysis was not possible with the included articles. We have presented results in the following categories: diet, physical activity, diet and physical activity combined, smoking, alcohol consumption, and multiple areas of behavior (all combinations other than diet and physical activity). Where there were enough reviews, we grouped by mode of delivery, especially internet (including email and interventions that require accessing a website) and mobile phone (including apps and SMS text messaging interventions); social media was categorized separately from internet, mainly because there are enough papers to make the subdivision worthwhile but also because the social aspect may differentiate social media interventions from other forms of internet interventions, so that it is appropriately considered a subclass [17]. When reporting effect sizes, for Cohen *d*, Hedges *g*, and other measures of standardized mean difference (SMD), we followed the convention that 0.2 is a small effect size, 0.5 is a medium effect size, and 0.8 is a large effect size [18]. For risk ratios (RRs), we classified 1.22 as small, 1.86 as medium, and 3.00 as large; for odds ratios (ORs), these were 1.32, 2.38, and 4.70, respectively [19].

## Results

### Overview

**Figure 1:**
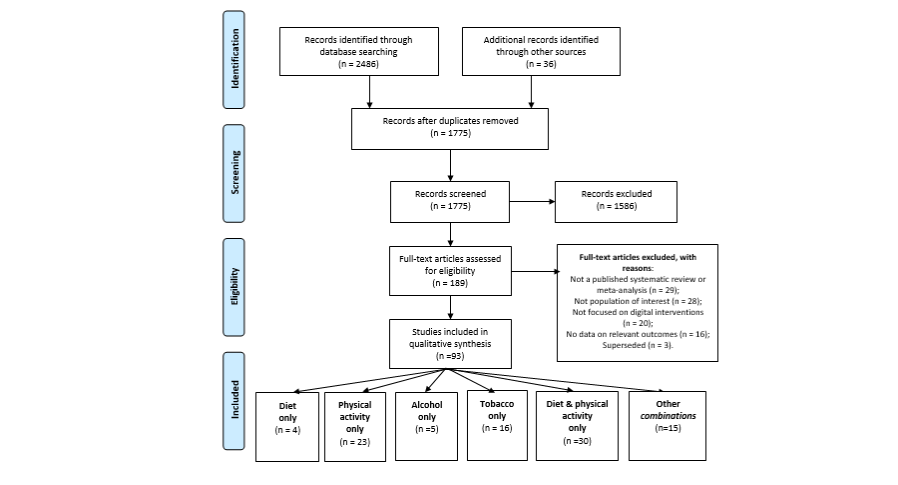
PRISMA flowchart. PRISMA: Preferred Reporting Items for Systematic Reviews and Meta-Analyses.

Searches identified 1739 potentially relevant records. After screening the titles and abstracts, 154 articles were retrieved in full. An additional 36 articles were identified through hand searches and gray literature searches. In total, 94 reviews met the inclusion criteria (Figure 1). A list of reviews excluded after full-text screening is provided in Multimedia Appendix 4. We were unable to retrieve one gray literature item by May 13, 2019, and it was therefore excluded.

### Review Characteristics

The included reviews examined the effectiveness of digital interventions on diet only [20-23], physical activity only [24-46], diet and physical activity combined [47-76] (for some it was possible to extract separate results about diet and physical activity behaviors, whereas some reported more general results on weight loss outcomes), alcohol consumption [77-82], and smoking cessation [83-98]. A further 15 reviews examined the effectiveness of digital interventions on a combination of our 4 target areas of behavior (not diet and physical activity; again, sometimes it was possible to extract separate information for each target area but other times, the results were only reported in combination) [99-113]. Some reviews covered a number of areas of behavior because their research questions focused on a health outcome (eg, CVD) or a mode of intervention delivery (eg, internet interventions) rather than a behavior. Where extracting information on an area of behavior from a combination review was possible, we included the relevant data in the results for that area. For a breakdown of the reviews, with the number found in each area, for each mode of intervention and type of control see Multimedia Appendix 5.

The populations reviewed were general (nonclinical) adult populations. However, the diet and physical activity reviews were often restricted to populations of individuals with overweight or obesity. The alcohol reviews were often restricted to *problem drinkers*, defined with reference to local guidelines [77,79,80], questionnaire scores [81], or reduced productivity at work [78]. There was a range of modes of delivery, including mobile app, SMS text messaging, social media, pedometer, wearable, and interactive computer program. The reviews included both active and nonactive or minimal intervention controls, many pooling both types, but where possible, we tried to extract separate information about effectiveness of active compared with nonactive controls. We regarded the provision of educational materials as a nonactive or minimal control. More reviews included behavioral outcomes (such as fat consumption, fruit and vegetable consumption, physical activity, alcohol consumption, smoking cessation, and smoking abstinence) than health outcomes (such as weight loss, BMI, and waist circumference). Multimedia Appendix 6 provides the key characteristics of all included studies.

### Review Confidence Ratings

The confidence rating of each review is presented in the study characteristics table in Multimedia Appendix 6, and a summary of confidence ratings is provided in Table 1; 84% (79/93) of reviews were rated as critically low. During the completion of the AMSTAR-2 tool, reviewers noted that most reviews failed to satisfy items 4 (including and justifying a publication language inclusion criterion) and 7 (providing a list of excluded reviews and justifications). Both items are considered critical for systematic reviews but not for meta-analyses. A modified rating was produced alongside the original AMSTAR-2 rating, which did not classify either of these flaws as critical, to see whether a variation among reviews would be revealed. However, little change was observed, with only 5 reviews moving from a rating of critically low to low.

**Table 1:** Risk of bias: a summary of AMSTAR-2 confidence ratings.

Category	Confidence rating (modified rating)			
	High	Moderate	Low	Critically low
Diet	0 (0)	0 (0)	1 (1)	3 (3)
Physical activity	0 (0)	1 (1)	0 (1)	20 (19)
Diet and physical activity	0 (0)	1 (1)	1 (3)	28 (26)
Alcohol	0 (0)	0 (0)	1 (1)	4 (4)
Smoking	0 (0)	2 (2)	4 (4)	10 (10)
Other	0 (0)	1 (1)	2 (4)	14 (12)
Total	0 (0)	5 (5)	9 (14)	79 (74)

### Effectiveness of Interventions

Table 2 summarizes the effectiveness of the different types of interventions.

**Table 2:** Summary of the effectiveness of different types of interventions.

Findings	Behavioral categories					
	Diet	Physical activity	Diet and physical Activity	Smoking	Alcohol	Other combinations
Number of reviews	20 reviews, of which 4 were diet-only	45 reviews, of which 22 were physical activity-only	35 reviews, of which 29 were on weight loss, specifically combining diet and physical activity, and 3 where results on weight loss were extractable from other combinations	28 reviews, of which 16 were smoking-only	13 reviews, of which 6 were alcohol-only	11
Effectiveness compared with mixed (active and nonactive) controls	Small effect of internet interventions on pooled behaviors but heterogenous outcome measures and effect sizes Some small effects of mobile phone interventions on behavior, but no effects on weight loss Mixed effects of social media but no effects on weight loss Mixed effects of computer-delivered interventions, and positive effects were not clinically significant	Mixed evidence for the effect of internet interventions on physical activity; where there were effects, they were small and there was heterogeneity between studies Mixed effects of social media, with meta-analyses not finding significant effects Better evidence for effects of mobile phone interventions, with most studies in narrative reviews showing effectiveness, with effect sizes for SMS text messaging interventions ranging from small to medium Mixed evidence for the effect of exergaming Mixed evidence on computer-delivered interventions (2 reviews); the meta-analysis found small effects There was favorable evidence from reviews assessing a variety or a combination of interventions	Positive effects of internet interventions for weight loss and BMI, especially when part of blended interventions Mixed effects of social media interventions Medium-sized effects of mobile interventions on weight loss Mixed results from reviews covering a variety of interventions, around half of the interventions were found to be effective	Small positive effects for internet, mobile, and computer-delivered interventions. Mixed evidence about interventions from reviews that covered multiple types of digital technologies	Small and medium positive effects	Small positive effects on behavior and on health outcomes for internet, computer-delivered, SMS text messaging, and prompts delivered by all methods; however, results were not favorable for apps
Comparison to no or minimal intervention	Little evidence available and it was mixed: One meta-analysis of voice-response interventions found no effect One meta-analysis of computer-tailored interventions found a small effect	Mixed results: Mixed results for internet and mobile interventions One review of social media interventions, finding increased in steps taken but not energy expenditure, total physical activity, or moderate-to-vigorous physical activity Small positive effect of interactive voice response and computer-delivered interventions	More effective than minimal controls for internet and computer-based interventions, but effect sizes were small	Small positive effects for internet-based interventions and for digital interventions not broken down by mode of delivery), mobile interventions more effective than completely passive controls and as effective as minimal controls	Effective compared with no-intervention controls	Effective compared with no-intervention controls, with small effects for internet and medium effects for SMS text messaging interventions
Comparison with active controls	Mixed; in narrative syntheses (of internet, mobile, and combinations of interventions), less than 50% of studies in each review found significant improvements	Increase in physical activity for internet-based interventions Mixed results for mobile interventions Promising results about wearables	Internet-based interventions not more effective than active controls, unless as a part of blended interventions Computer-delivered interventions led to less weight loss than in-person treatment	No evidence that digital interventions are any more effective than active controls	No evidence that digital interventions are any more effective than active controls; mixed evidence about whether active controls are more effective than digital	Very small effects for internet and small effects for computer-delivered and SMS text messaging interventions
Sustainability	Relatively few studies with follow-ups Digital interventions were largely effective over a 3- to 6-month period Evidence was more mixed in the long term (>12 months)	Relatively few studies with follow-ups Some evidence for sustainability of internet and combinations of digital interventions at 6-month follow-up Some evidence that the effect of mobile interventions is short lived	Few studies, but those found that effectiveness declined over time	Mixed evidence: Effects of internet-based interventions sustained up to 6 months, but there is disagreement about whether effects are maintained at the 12-month follow-up Mixed evidence on the sustainability of mobile interventions No evidence of sustainability of computer-delivered interventions Reviews that surveyed a range of digital interventions without differentiating by mode of delivery found sustained effects, even at the 18-month follow-up	Mixed results about whether the effect was sustained. No follow-ups exceeded 12 months. Evidence suggests effects have diminished by this point	Mixed evidence on when the effect size peaks (short or medium term); effects decrease but still exist at 12 months

#### Diet

##### Review Characteristics

A total of 20 reviews reported findings on diet behaviors or a weight loss outcome that resulted from an intervention that only targeted diet. The breakdown of their characteristics is shown in Table S1.

##### Effectiveness of Digital Interventions on Diet Compared With Mixed (Active and Nonactive) Controls for Diet Behaviors

The effectiveness of digital interventions in improving results related to diet was at best mixed, for both behavioral and health outcomes (Table 3); where improvements were reported, the effect sizes were typically small. This was the case across all modes of delivery.

**Table 3:** Results of reviews on diet, ordered by type of control and mode of delivery of intervention.

Review		Relevant studies, n (total studies)		Method of synthesis	Interventions	Dietary outcomes		Follow-up		Summary of findings	AMSTAR-2 rating
**Mixed (active and nonactive) controls**											
	Afshin et al (2016) [99]		20 (224)	Narrative synthesis of RCTs^a^ and quasiexperimental studies	Internet and mobile		Various dietary behaviors, fruit and veg intake		1 week-2 years	70% (14/20) studies found significant dietary improvements. Effect sizes varied due to heterogeneity in dietary targets. The intake of fruit increased by approximately 1 serving/day. Two RCTs assessed mobile‐based interventions and fruit or vegetable intake; each found significant improvement (by 2 and 4 servings/day)	Critically low
	Aneni et al (2014) [100]	9 (29)		Narrative synthesis of RCTs	Internet	Dietary intake		6-24 months		44% (4/9) high-quality studies demonstrated improvements in diet	Critically low
	Hou et al (2013) [108]	7 (38)		Narrative synthesis of studies with comparison or control groups	Internet	Dietary intake and fruit and vegetable intake		Not reported		All the studies examining nutrition alone and nutrition and other factors in addition to PA^b^ reported increases in healthy dietary behaviors in the intervention groups. Interventions showed promising effects at 12 months for reductions in body fat, weight, and dietary fat intake	Critically low
	Lustria et al (2013) [110]	10 (40)		Meta-analysis of experimental and quasiexperimental studies	Internet-tailored interventions	Fruit and vegetable intake and saturated fat intake		6 weeks-6 months		Small effect sizes, Cohen *d*=0.223 (k=4; 95% CI 0.11 to 0.33; *P*<.001)	Critically low
	Maon et al (2012) [63]	8 (26)		Narrative synthesis of RCTs	Internet	Fruit and vegetable consumption		6 weeks-2 years		50% (4/8) studies investigating healthy eating habits reported positive changes such as increased fruit or vegetable consumption	Critically low
	Webb et al (2010) [113]	10 (85)		Meta-analysis of RCTs	Internet	Dietary behavior		Not reported		Small effect sizes on behavior were observed for interventions that targeted only dietary behavior (Cohen *d*_+_=0.20; k=10; 95% CI 0.02 to 0.37)	Critically low
	DiFillipo et al (2015) [21]	3 (3)		Narrative synthesis of RCTs	Mobile	Weight loss		8 weeks-6 months		100% (3/3) of studies found a numerical tendency to weight loss compared with the control; only 33% (1/3) of studies was statistically significant; this study showed an increase in self-monitoring	Critically low
	McCarroll et al (2017) [23] (diet only)	21 (23)		Narrative synthesis of RCTs	Mobile	Healthy eating and weight loss (diet only)		1-24 months		Small positive effects of interventions on healthy eating (5/8, 63% of trials) and weight loss (5/13, 38% of trials), but studies were judged to be of poor quality	Critically low
	Lyzwinksi et al (2014) [60]	5 (14)		Meta-analysis of RCTs	Mobile	Fruit and veg intake, energy density		8 weeks-12 months		100% (3/3) of studies found an improvement in fruit and vegetable intake; 100% (2/2) studies found an improvement in energy density and eating behavior	Critically low
	Palmer et al (2018) [111]	3 (71)		Meta-analysis of RCTs	Mobile	Saturated fat intake, BMI, salt intake, weight from diet-only interventions		Not reported		2 (100%) studies showed no effect of interventions on weight or dietary intake	Moderate
	Elaheebocus et al (2018) [56]	20 (134)		Narrative synthesis of RCTs	Social media	Body weight		6-48 months		75% (15/20) of studies using web-based social networks had positive results for dietary outcomes	Critically low
	Mita et al (2016) [65]	12 (16)		Meta-analysis of RCTs	Social media	Body weight and fruit and vegetable intake		Not reported		No significant differences (SMD^c^ −0.14; 95% CI −0.28 to 0.01), with similar findings for body weight (SMD 0.07; 95% CI −0.17 to 0.20) and fruit and vegetable intake (SMD 0.39; 95% CI −0.11 to 0.89)	Moderate
	Williams et al (2012) [75]	5 (22)		Meta-analysis of RCTs	Social media	Body weight and dietary fat		3-24 months		No significant differences in changes in weight (SMD 0; 95% CI −0.19 to 0.19; 10 studies) however, pooled results from 5 studies showed a significant decrease in dietary fat consumption with social media (SMD −0.35; 95% CI −0.68 to −0.02)	Critically low
	Wieland et al (2012) [74]	18 (18)		Meta-analysis of RCTs, quasi-RCTs, cluster RCTs, and quasiexperimental studies	Interactive, computer-based	Fat intake, calorie intake, total fiber, fruit and vegetable intake; weight, BMI, and waist circumference		4 weeks-30 months		Computer-based interventions led to greater weight loss than minimal interventions (MD^d^ −1.5 kg; 95% CI −2.1 to −0.9) but less weight loss than in-person treatment (MD 2.1 kg; 95% CI 0.8 to 3.4; one trial); in the 3- to 6-month follow-up, there was a significant decrease in percentage calories from fat (MD −1.1%), and improved fiber intake (dietary fiber score MD 1.3); there was no significant effect on energy intake at 30 months	Critically low
	Harris et al (2011) [22] (diet only)	25 (43)		Meta-analysis of RCTs	Interactive, computer based	Fat intake, fruit and vegetable consumption, fiber intake, and energy intake		0-12 months		Interventions did not produce clinically significant changes in dietary behavior: fruit and vegetable intake had a weighted MD of 0.24 servings per day (95% CI 0.04 to 0.44 servings; *P*=.02), total energy consumed from fat had a weighted MD of –1.4% (95% CI –2.5% to –0.3%; *P*=.01); there were no significant weighted MDs in intake of total fat, saturated fat, daily dietary fiber, or daily energy; there were no significant effects on fruit and vegetable consumption and BMI at 12 months	Low
	Carvalho de Menzes et al (2016) [53]	15 (18)		Narrative synthesis of all designs	Various	Fat consumption and fruit and vegetable consumption		1-36 months		Among the changes in eating habits, statistically significant reductions were observed in the consumption of fat (total fat, saturated fat, and transfat), and there was an increase in the ingestion of fruit and vegetables	Critically low
**Nonactive controls**											
	Krebs et al (2010) [109]	51 (76)		Meta-analysis of RCTs	Computer-tailored	Dietary intake of fat, vegetables, and fruits		1-24 months		The mean effect size for dietary fat reduction was *g*=0.22 (95% CI 0.18 to 0.26); mean effect size for fruit and vegetable intake was *g*=0.16 (95% CI 0.10 to 0.21).	Low
	Tsoli et al (2018) [73]	2 (15)		Meta-analysis of RCTs	Interactive voice response	Diet		6 weeks-12 months		No statistically significant effect on behaviors related to diet (Hedges *g*=0.130; 95% CI −0.088 to 0.347; k=2; *P*=.24)	Critically low
**Active controls**											
	Burke et al (2011) [20] (diet only)	5 (24)		Narrative synthesis of RCTs and descriptive studies	Personal digital assistants	Weight loss (diet only)		3-24 months		In all studies, self-monitoring was significantly associated with weight loss, but there was minimal evidence to support the use of personal digital assistants over other methods of self-monitoring; they may result in long-term reduced risk of weight regain after 18 months	Critically low
	Covolo et al (2017) [104]	18 (40)		Narrative synthesis of RCTs	Mobile	BMI and waist circumference; fruit and vegetable intake, and high-sugar food intake		6 months-2 years		56% (10/18) of RCTs found no difference between intervention and control group; 33% (6/18) studies showed a significant increase in the consumption of fruit and vegetables and reduced sugar-sweetened beverage consumption	Critically low

^a^RCT: randomized controlled trial.

^b^PA: physical activity.

^c^SMD: standardized mean difference.

^d^MD: mean difference.

Of the 6 reviews covering internet interventions, 2 meta-analyses found small, favorable effects of internet interventions on dietary behavior, Cohen *d*=0.223 [110] and Cohen *d+*=0.20 [113]. A total of 4 narrative syntheses found mixed results with heterogeneous target outcomes [63,99,100,108]. The most common dietary target across the studies was fruit intake, which increased by approximately 1 serving per day [99].

Across 6 reviews of mobile interventions, there was some evidence of positive effects on dietary behaviors, especially fruit and vegetable intake, but no statistically significant effects on weight loss [21,23,60,99,104,111]. One of these reviews [99] reported a previous systematic review (not covered in this paper) that found no significant change in calorie intake or the consumption of sugar-sweetened beverages in 4 trials evaluating diet.

A total of 3 social media reviews had mixed findings on dietary outcomes, such as fruit and vegetable intake and fat intake, but the 2 reviews with meta-analyses found no differences in weight [65,75].

The effects of interactive computer interventions were equivocal and not clinically significant [22,74].

##### Effect of Digital Interventions on Diet Compared With Nonactive Controls

There was little evidence about the effectiveness of digital interventions on diet compared with no intervention or minimal intervention controls, and the available evidence was mixed (Table 3). One meta-analysis found small effects on fruit and vegetable intake (*g=*0*.*16) and dietary fat reduction (*g=*0*.*22) [109], but another meta-analysis found no statistically significant effects on dietary behaviors [73].

##### Effect of Digital Interventions on Diet Compared With Active Controls

The relative effectiveness of digital interventions on diet compared with active comparators was mixed, with approximately half of the studies or less in narrative syntheses, showing that digital interventions were effective compared with active controls (Table 3). This was true across all modes of delivery: internet [99], mobile [99,104], and combined interventions [99].

##### Sustainability of Effects on Diet at Follow-Up

Reviews included studies that ranged from single-contact interventions to 5-year follow-ups (Table 3). Relatively few reviews reported on follow-ups; of those that did, about half reported follow-ups in the medium term (3-6 months) and half in the long term (≥12 months).

Where reported, digital interventions were generally found to be effective for over 3 to 6 months. Several reviews have found positive results at 6 months [20,21,74]. However, this finding is not universal [111].

Long-term findings were more mixed in the 5 reviews that investigated them. Two reviews suggested promising effects at 12 months [108] and 18 months [20]. However, 3 reviews found no significant effects at 12 months [22], 24 months [111], and 30 months [74].

#### Physical Activity

##### Review Characteristics

We included the findings on physical activity–related outcomes from 45 systematic reviews. The breakdown of their characteristics is shown in Multimedia Appendix 6.

##### Effectiveness of Digital Interventions on Physical Activity Compared With Mixed (Active and Nonactive) Controls

The effectiveness of digital interventions was mixed across all modes of delivery, apart from mobiles, for which the evidence was consistently positive (Table 4).

**Table 4:** Results of reviews on physical activity, ordered by type of control and further ordered by mode of delivery of intervention.

Review			Relevant studies, n (total studies)		Method of synthesis		Interventions		Outcomes		Follow-up		Summary of findings		AMSTAR-2 rating
**Mixed controls (active and nonactive)**															
	Aalbers et al (2011) [47]	2 (10)		Narrative synthesis of randomized and nonrandomized pre-post controlled trials		Internet		Total PA^a^ and MVPA^b^		1.5-6.5 months		2 studies reported the effects of digital interventions, one was less effective on MVPA than a nonactive control and the other demonstrated a small positive effect on total PA^b^ (*P*=.001).		Critically low	
	Aneni et al (2014) [100]	9 (29)		Narrative synthesis of RCTs^c^		Internet		PA measures		6-24 months		No improvement was seen in virtually all the studies with PA outcome, only 11% (1/9) of studies demonstrated a significant intervention effect on PA.		Critically low	
	Bottorf et al (2014) [26] (PA only)	8 (35)		Narrative synthesis of all study types		Internet		Changes in PA; step count; self-reported walking; BMI; waist circumference; weight		3-12 months		63% (5/8) of studies demonstrated that PA significantly increased in the internet-based interventions, 2 studies showed a nonsignificant difference, and one showed that the effects were indeterminable.		Critically low	
	Davies et al (2012) [29] (PA only)	34 (34)		Meta-analysis of experimental design studies		Internet		PA		2-52 weeks		The estimated overall mean effect of internet-delivered interventions on PA was Cohen *d*=0.14 (*P*<.001). Homogeneity tests from the fixed-effect analysis revealed significant heterogeneity across studies (*Q*=73.75; *P*<.001). The overall mean effect for sustained PA at least 6 months postintervention (n=11) resulted in a small but significant effect size Cohen *d*=0.11 (*P*<.01).		Critically low	
	George et al (2012) [33] (PA only)	2 (14)		Narrative synthesis of all study types		Internet		Step count; health status; BMI; weekly PA		2-8 months		Increase in PA in 100% (2/2) of online interventions where participants were in competitive teams, including one that showed an increase in step count. Poor quality evidence.		Critically low	
	Hou et al (2013) [108]	7 (38)		Narrative synthesis of trials with comparison or control group		Internet		Level of physical activity		0-12 months		86% (6/7) of interventions were successful in the studies focusing primarily on PA.		Critically low	
	Jahangiry et al (2017) [35] (PA only)	21 (22)		Meta-analysis of controlled trials		Internet		MVPA; walking; step count (pedometer)		1-20 weeks		36% (5/14) of rials reporting MVPA, 50% (3/6) of trials reporting step count, and 29% (4/14) of studies reporting minutes walking showed significant increases. The interventions were influenced by the age of participants and trial length.		Critically low	
	Lustria et al (2013) [110]	12 (40)		Meta-analysis of experimental and quasiexperimental studies		Internet		Levels of PA		4 weeks-24 months		The sample size–weighted mean effect size for studies on PA was not significant Cohen *d*=0.059 (k=12; 95% CI −0.02 to 0.14).		Critically low	
	Maon et al (2012) [63]	13 (26)		Meta-analysis and narrative synthesis of RCTs		Internet		PA levels, sedentary behavior, and MVPA		6 weeks-2 years		54% (7/13) of studies showed statistically significant effects on PA levels, such as increased walking or decreased sedentary behavior. However, a meta-analysis on 4 studies with extractable data for the outcome of moderate-to-vigorous weekly PA found a not statistically significant improvement: SMD^d^ 0.15 (95% CI 20.06 to 0.35; *P*=.16) Duration of studies and effects: 10% (3/30) of studies showed positive effects when outcomes were measured immediately after the end of the interventions. In total, 37% (11/30) of studies that lasted 3 months or less demonstrated positive outcomes; 43% (13/30) of studies with an intervention of 3-6 months showed positive results; and only 10% (3/30) interventions that lasted longer than 6 months were reported to have positive results.		Critically low	
	Webb et al (2010) [113]	20 (85)		Meta-analysis of RCTs		Internet		Level of PA		3-12 months		Small effects on behavior were observed for interventions that targeted only PA (Cohen *d*+=0.24; k=20; 95% CI 0.09 to 0.38).		Critically low	
	Buchholz et al (2013) [27] (PA only)	10 (10)		Narrative synthesis of RCTs, quasiexperimental and, single groups		Mobile (SMS text messaging)		Self-reported frequency or pedometer-reported steps and level of PA		3-52 weeks		Effect sizes across all studies were positive; the median effect size was 0.5 (medium) but heterogeneous. Sample sizes were small.		Critically low	
	Elavsky (2018) [31] (PA only)	50 (52)		Narrative synthesis of RCTs and pre-post studies		Mobile		PA and sedentary behavior		<3 months		59% (17/29) of RCTs and 62% (13/21) of pre-post studies supported the effectiveness of mobile interventions to improve PA, and 9 (5 of 10 RCTs and all 4 pre-post) of 14 (64%) studies reduced sedentary behavior.		Critically low	
	Lyzwinksi et al (2014) [60]	9 (14)		Meta-analysis of RCTs		Mobile		Levels of PA		8 weeks-12 months		Trials mostly found that PA levels increased in the intervention groups relative to the control groups.		Critically low	
	Maher et al (2014) [61]	4 (10)		Narrative synthesis of studies with comparator group (control or within subject)		Mobile		Levels of PA		8 weeks-24 months		25% (1/4) of studies demonstrated a significant change in PA Cohen *d=*0.84 (95% CI −0.49 to 1.19).		Critically low	
	Muntaner et al (2015) [41] (PA only)	11 (11)		Narrative synthesis of all study types		Mobile		PA; exercise		2-24 weeks		55% (6/11) of articles included in this review reported significant increases in PA levels.		Critically low	
	O'Reilly et al (2013) [42] (PA only)	12 (22)		Narrative synthesis of RCTs		Mobile		PA; sedentary behavior; BMI; blood lipids; blood pressure; QoL^e^; adverse effects		Not reported		75% (9/12) of studies reported significant changes in PA or sedentary behavior.		Critically low	
	Palmer et al (2018) [111]	15 (71)		Meta-analysis of RCTs		Mobile		Level of PA		3 months		Trials of PA interventions reporting outcomes at 3 months showed no benefits.		Moderate	
	Schoeppe et al (2016) [68]	10 (27)		Narrative synthesis of RCTs, randomized trials, controlled trials, and pre- and poststudies		Mobile		PA; sedentary behavior		1-24 weeks		59% (13/22) of studies reported significant improvements in levels of PA; 20% (1/5) of studies reported a significant change in sedentary behavior.		Critically low	
	Elaheebocus et al (2018) [56]	25 (134)		Narrative synthesis of RCTs		Social media		Body weight		6-48 months		76% (19/25) of studies using online social networks had positive results.		Critically low	
	Mita et al (2016) [65]	11 (16)		Meta-analysis of RCTs		Social media		PA; weight change		1-12 months		For PA, significant mean difference 0.07; 95% CI −0.25 to 0.38; k=11.		Moderate	
	Williams et al (2012) [75]	12 (22)		Meta-analysis of RCTs		Social media		PA		3-24 months		Meta-analysis showed no significant differences in changes in PA (SMD 0.13; 95% CI −0.04 to 0.30; k=12).		Critically low	
	Willis et al (2017) [76]	3 (5)		Narrative synthesis of all study types		Social media		Total PA		8 weeks-6 months		Only one study reported significant changes in levels of PA, when the web-based social network intervention included an online support group.		Critically low	
	Johnson (2017) [37]	10 (19)		Narrative synthesis of RCTs		Active gaming		Behavioral and cognitive outcomes		Not reported		Findings were largely positive for behavioral impacts, specifically the impact of gamification for PA: 80% (8/10) positive and 20% (2/10) mixed.		Critically low	
	Peng et al (2012) [43] (PA only)	4 (12)		Narrative synthesis of all study designs		Active gaming		Heart rate; energy expenditure; and oxygen uptake		6-12 weeks		Evidence does not support active video games as an effective tool to significantly increase PA or exercise attendance.		Critically low	
	Street et al (2017) [46] (PA only)	9 (9)		Narrative synthesis of studies with comparison or control groups		Active gaming		PA; maximum oxygen uptake; power; blood pressure; body mass; body weight; body fat; BMI; balance; speed; and strength		6-12 weeks		Moderate-to-high exergaming participation was associated with statistically significant improvements in anthropometric outcomes but low participation was not associated with anthropometric changes. 38% (3/8) studies that investigated anthropometric outcomes, including BMI and body fat, found a statistically significant improvement, all 3 studies showed positive health outcomes associated with moderate-to-high participation in exergaming; 100% (3/3) of studies that reported on PA frequency reported higher frequency in the exergaming condition; however, a different 100% (3/3) of studies that reported on overall PA found no statistically significant increases.		Critically low	
	Wieland et al (2012) [74]	4 (18)		Meta-analysis of RCTs, quasi-RCTs, cluster RCTs, and quasiexperimental studies		Computer-delivered		Steps per day and minutes walked continuously		4 weeks-30 months		No studies demonstrated statistically significant effects on PA.		Critically low	
	Afshin et al (2016) [99]	33 (224)		Narrative synthesis of RCTs and quasi-experimental studies		Various: internet and mobile		Level of PA		1 week-5 years		88% (29/33) of studies reported significant improvement in PA; 83% (5/6) of phone interventions were effective, including 66% (2/3) of SMS text messaging interventions, 100% (2/2) of apps, and 100% (1/1) of automated voice response.		Critically low	
	Carvalho de Menzes et al (2016) [53]	13 (18)		Narrative synthesis of all study designs		Various: email, telephone, websites		Level of PA		1-36 months		Most studies demonstrated statistically significant improvements in the level of PA.		Critically low	
	Hakala et al (2017) [34] (PA only)	13 (23)		Meta-analysis of RCTs		Various: mobile, text messages, pedometers, wearables, email		PA: self-reported or using an accelerometer or pedometer		3 weeks-24 months		No differences were observed between the experimental and control groups (risk ratio 1.03; 95% CI 0.92 to 1.15; *P*=.57).		Critically low	
	Muellmann et al (2018) [39] (PA only)	13 (20)		Narrative synthesis of experimental designs and quasiexperimental studies		SMS text messaging and internet		PA and number of steps per day		4 weeks-24 months		75% (3/4) of studies using mobile phones demonstrated significant differences in the level of PA or steps per day (mixed controls). In 100% (9/9) of studies, internet interventions significantly increased PA compared with nonactive controls.		Critically low	
	Muller and Khoo (2014) [40] (PA only)	4 (16)		Narrative synthesis of RCTs and quasiexperimental studies		Various: internet and mobile		PA		1 week-18 months		75% (3/4) of studies reported significant improvements in PA; 25% (1/4) of studies reported nonsignificant decrease in PA.		Critically low	
	Stephenson et al (2017) [45] (PA only)	15 (17)		Meta-analysis of RCTs		Various: mobile messaging, mobile apps, website, wearable technology		Sedentary behavior		5 days-24 months		Interventions using computer and mobile and wearable technologies can be effective in reducing sedentary behavior. Effectiveness appeared most prominent in the short-term and lessened over time. Meta-analysis of 88% (15/17) of RCTs suggested that computer, mobile, and wearable technology tools resulted in a mean reduction of −41.28 min/day of sitting time (95% CI −60.99 to −21.58; I^2^=77%). The pooled effects showed mean reductions at short (≤3 months), medium (>3 to 6 months), and long-term (>6 months) follow-up of −42.42 min/day, −37.23 min/day, and −1.65 min/day, respectively.		Critically low	
**Nonactive controls**															
	Jenkins et al (2009) [36] (PA only)	5 (22)		Narrative synthesis of RCTs		Internet		PA		0-24 months		Results were mixed; internet interventions can be effective, compared with control conditions, although poor compliance was an issue. 50% (2/4) studies reported an increase in PA compared with nonactive controls while 2 studies found no difference.		Critically low	
	Bock et al (2014) [24] (PA only)	4 (50)		Narrative synthesis of RCTs and quasiexperimental studies		Internet or computer		Weekly PA, proportion of sufficiently active persons; step counts		0 weeks-3 years		Interventions had a nonsignificant, positive effect on PA (*P*=.88).		Critically low	
	Krebs et al (2010) [109]	25 (76)		Meta-analysis of RCTs		Computer-delivered		Minutes of PA		1-18 months		The mean effect size was *g*=0.16 (95% CI 0.10 to 0.21).		Low	
	Bort-Roig et al (2014) [25] (PA only)	5 (26)		Narrative synthesis of comparative and pre-postdesign		Mobile		PA (steps); energy expenditure; body weight and body fat; blood pressure and cholesterol; QoL		2 weeks-6 months		80% (4/5) of studies assessing PA intervention effects reported PA increases, with mean PA increases ranging from 800 to 1104 steps/day. Studies were small with differences in baseline characteristics.		Critically low	
	Direito et al (2017) [30] (PA only)	17 (21)		Meta-analysis of RCTs		Mobile		PA, MVPA, walking and sedentary behavior		1-52 weeks		Not effective for MVPA outcomes, based only on adult studies SMD 0.14 (95% CI −0.10 to 0.37). For sedentary behavior outcomes, SMD −0.21 (95% CI −0.59 to 0.18).		Critically low	
	Freak-Poli et al (2013) [32] (PA only)	4 (4)		Narrative synthesis of RCTs and cluster RCTs		Wearable technology		PA; sedentary behavior; BMI; blood lipids; blood pressure; QoL; adverse effects		3-8 months		Overall, there was insufficient evidence to assess the effectiveness of pedometer interventions in the workplace. 75% (3/4) of studies compared with a minimal control group, 33% (1/3) of studies observed an increase in PA under a pedometer program, but the other two did not find a significant difference.		Moderate	
	An et al (2017) [49]	21 (22)		Meta-analysis of RCTs, pre-post studies, and cohort studies		Social media		PA, sedentary behavior		3-102 weeks		Interventions increased daily number of steps taken by 1530 (95% CI 82 to 2979). However, they were not associated with energy expenditure, total PA, or MVPA.		Critically low	
	Tsoli et al (2018) [73]	3 (15)		Meta-analysis of RCTs		Interactive voice responses		PA		6 weeks-12 months		Interventions led to a small but statistically significant increase in PA (*g*=0.254; 95% CI 0.068 to 0.439; k=3; *P*=.007).		Critically low	
**Active controls**															
	Beishuizen et al (2016) [102]	5 (57)		Meta-analysis of RCTs		Internet		Level of PA		4 weeks-3 months		Interventions led to an increase in PA (SMD 0.25; 95% CI 0.10 to 0.39).		Low	
	Covolo et al (2017) [104]	23 (40)		Narrative synthesis of RCTs		Mobile apps		Daily steps, frequency, and intensity of PA		6 months-2 years		30% (7/23) of RCTs showed a significant increase in PA in the intervention group (measured in daily steps, frequency of PA, or level of intensity), 48% (11/23) of studies did not show a significant increase, and in 21% (5/23) studies, outcome measures were inconsistent in whether there was a significant difference between intervention and control.		Critically low	
	Mateo et al (2015) [38] (PA only)	10 (11)		Meta-analysis of controlled trials		Mobile apps		PA, MVPA, and steps		6 weeks-9 months		Compared with the control group, use of a mobile phone app was associated with significant changes in body weight and BMI of −1.04 kg (95% CI −1.75 to −0.34; I^2^=41%) and −0.43 kg/m^2^ (95% CI −0.74 to −0.13; I^2^=50%), respectively (k=9); however, a nonsignificant difference in PA was observed between the intervention and comparison groups (SMD 0.40; 95% CI −0.07 to 0.87; I^2^=93%).		Critically low	
	Song et al (2018) [44] (PA only)	6 (8)		Narrative synthesis of all study types		Mobile		PA (frequency and step count); BMI; blood glucose		4 weeks-6 months		Significant effects on frequency of PA in 80% (4/5) of studies (though the effect was reported to have disappeared after the 12-week follow-up), step count in 66% (2/3) of studies, BMI in 50% (2/4) of studies, and reduction in glucose in 100% (2/2) studies.		Critically low	
	Cheatham et al (2018) [28] (PA only)	25 (25)		Narrative synthesis of controlled clinical trials		Wearable technology		PA; BMI; weight; blood pressure; Resting Energy Expenditure; body composition; cardiovascular fitness; work productivity and absenteeism; waist circumference; blood parameters		3 weeks-24 months		An activity tracker combined with a comprehensive weight loss program may provide superior short-term (≤6 months) results than a standard weight loss program in middle aged or older adults. 80% (20/25) of studies reported higher weight loss when an activity tracker was used with a weight loss intervention.		Critically low	

^a^PA: physical activity.

^b^MVPA: moderate-to-vigorous physical activity.

^c^RCT: randomized controlled trial.

^d^SMD: standardized mean difference.

^e^QoL: quality of life.

Evidence for the effectiveness of internet interventions on physical activity has been mixed. In total, 5 out of 10 reviews were positive [26,29,33,108,113], including 2 meta-analyses that found small but significant effects of internet interventions: Cohen *d*=0.14 [29] and Cohen *d*+=0.24 [113]. However, there was significant heterogeneity across studies [29]. In contrast, 5 studies were not positive [35,47,63,100,110], one of which was very unfavorable, with only 1 of 9 (11%) studies demonstrating an effect of the intervention [100]. Two meta-analyses found that the effect of internet interventions on physical activity was not significant [63,110].

A total of 4 reviews of social media interventions were mixed. In total, 2 meta-analyses of social media interventions found no significant difference in changes in physical activity [65,75], and a narrative synthesis reported mixed results [76]. However, one narrative synthesis found that 76% of studies using web-based social networks had positive results for physical activity [56].

The results of the mobile interventions to improve health were more positive. In total, 8 of 10 (80%) narrative syntheses reported a majority of positive results [25,27,31,39,41,42,60,99], with one reporting an increase of 800 to 1104 steps per day [25]. One review noted that effective interventions used SMS text messaging communication or self-monitoring [42]. A review that was specifically on SMS text messaging reported that effect sizes were all greater than 0.20, and the median was 0.50, a medium effect size [27]. In contrast, 2 narrative syntheses reported that most mobile trials did not show any benefits [61,111].

There was mixed evidence of active gaming across 3 reviews. One review found that gamification has a positive impact on physical activity and found evidence that gamification can increase motivation to exercise [37]. However, another review found a positive effect on attendance but not on physical activity or BMI [46]. A third review found that active gaming did not support increases in either physical activity or attendance [43].

Computer-delivered interventions in physical activity behaviors did not have consistent results in either weight loss or weight maintenance trials [74].

In total, 4 of the 5 reviews assessing a variety of interventions found favorable results [40,45,53,99]. One of the narrative syntheses reported a wide range of values for improvement, from 1.5 to 153 extra minutes of physical activity a week and 1000 to 2600 steps per day [99]. A meta-analysis found that computer, mobile, and wearable technology led to a mean change of −41.28 minutes per day of sitting time (a reduction in sitting time) [45]. However, one meta-analysis found no difference between the experimental and control groups [34].

##### Effectiveness of Digital Interventions on Physical Activity Compared With Nonactive Controls

Compared with minimal controls, evidence for the effectiveness of digital interventions has been mixed.

For internet interventions, one review found favorable evidence [39], another found unfavorable evidence [24], and a third found mixed evidence [36].

The 3 reviews of mobile interventions have also provided mixed evidence. One narrative synthesis found that interventions were effective, with 4 studies (3 pre-post and 1 comparative) reporting increases of 800 to 1104 steps per day [25]. However, another study found that mobile interventions were not effective in increasing physical activity of moderate-to-vigorous intensity or in decreasing sedentary behavior [30]. Wearables were also not very effective, with only 1 of 3 (33%) studies comparing a pedometer with a minimal control showing increased physical activity [32].

Social media–based interventions increased the daily number of steps taken by 1530 steps per day [49]. However, they were not associated with energy expenditure, total physical activity, or moderate-to-vigorous physical activity.

There were small effect sizes for both computer-delivered interventions (*g*=0.16) [109] and interactive voice response–based interventions (*g*=0.254) [73].

##### Effectiveness of Digital Interventions on Physical Activity Compared With Active Controls

There were mixed results compared with active controls.

A meta-analysis of internet interventions found an increase in physical activity with an SMD of 0.25 compared with active controls [102].

In total, 3 reviews of mobile phones had active controls, and there were mixed results. A meta-analysis found that the use of a mobile phone app was associated with significant changes in body weight (−1.04 kg) and BMI (−0.43 kg/m^2^); however, there was no significant difference in physical activity between the 2 groups [38]. A narrative synthesis app was also not favorable for assessing changes in physical activity, with less than half of the studies showing a significant increase in physical activity in the intervention group [104]. However, another narrative synthesis of general mobile interventions found that most studies had interventions that led to changes in body weight, increases in step count, and increases in frequency of physical activity [44].

There were also promising results from a review on wearables: when an activity tracker is combined with a comprehensive weight loss program, it may provide superior short-term (≤6 months) results than a standard weight loss program in middle-aged or older adults (>30 years) [28].

##### Sustainability of Effects on Physical Activity at Follow-Up

There was little evidence on sustainability, as many physical activity studies did not have follow-up assessment postintervention or only had follow-ups relatively soon after the intervention end point.

There is some evidence that digital interventions can have sustained effects. A meta-analysis assessing combinations of digital technologies found that the pooled effects showed mean changes (reductions) at short (≤3 months), medium (3 to 6 months), and long-term follow-up (>6 months) of −42.42 minutes per day, −37.23 minutes per day, and −1.65 minutes per day, respectively [45]. A meta-analysis of internet interventions also found a small but significant effect on physical activity for follow-ups at least six months postintervention (Cohen *d*=0.11) [29]. The sustainability of internet interventions was also supported by a narrative synthesis that found that only 12 of 35 (34%) studies had follow-up assessments, which ranged from 7 weeks to 15 months postprogram; 10 out of 12 (83%) studies demonstrated successful maintenance of physical activity and/or secondary measures indicative of positive changes in physical activity; however, follow-up durations were primarily shorter: in 9 studies, follow-up was conducted at less than 12 months [26]. However, for mobile interventions, 2 reviews found evidence that effects tended to decrease in the long term [31], with effects disappearing after as little as 12 weeks [44].

#### Diet and Physical Activity (Weight Management)

##### Review Characteristics

A total of 35 reviews reported on both diet and physical activity. The breakdown of their characteristics is shown in Multimedia Appendix 6.

##### Effectiveness of Digital Interventions on Diet and Physical Activity (Weight Loss) Compared With Mixed (Active and Nonactive) Controls

Overall, digital interventions were generally found to be effective, with mobile phone interventions in particular having consistently positive results (Table 5).

**Table 5:** Results of reviews on diet and physical activity combined, ordered by type of control and further ordered by mode of delivery of intervention.

Review		Relevant studies, n (total studies)	Method of synthesis	Interventions	Outcomes	Follow-up	Summary of findings	AMSTAR-2 rating
**Mixed (active and nonactive) controls**								
	Aalbers et al (2011) [47]	5 (10)	Narrative synthesis of RCTs^a^ and nonrandomized pre-post controlled trials	Internet	Body weight and body weight regain	1.5-6.5 months	40% (2/5) of studies reported effect sizes on body weight with small-to-medium significant effects; 1 study reported weight regain but did not reach significance.	Critically low
	Aneni et al (2014) [100]	20 (29)	Narrative synthesis of RCTs	Internet	Weight, BMI, waist circumference, and body fat	6-24 months	Modest improvements were observed in more than half of the studies with weight-related outcomes; 20 studies reported on body weight: 75% (15/20) of high quality and 5 of 20 (25%) low quality); 47% (7/15) high-quality studies reported significant improvement.	Critically low
	Beishuizen et al (2016) [102]	7 (57)	Meta-analysis of RCTs	Internet	Systolic blood pressure, diastolic blood pressure, HbA_1c_^b^ level, cholesterol level, weight, and level of physical activity	3-60 months	There was a significant reduction in systolic blood pressure (MD^c^ –2.66 mm Hg; 95% CI –3.81 to –1.52), diastolic blood pressure (MD –1.26 mm Hg; 95% CI –1.92 to –0.60), HbA_1c_ level (MD –0.13%; 95% CI –0.22 to –0.05), LDL^d^ cholesterol level (MD –2.18 mg/dL; 95% CI –3.96 to –0.41), weight (MD –1.34 kg; 95% CI –1.91 to –0.77), and an increase in physical activity (SMD^e^ 0.25; 95% CI 0.10 to 0.39).	Low
	Fry et al (2009) [57]	8 (19)	Narrative synthesis of all study types	Internet	Diet and physical activity	8 weeks-30 months	There were generally positive effects of prompts; there was not enough evidence to know whether the medium in which prompts were sent through affected their effectiveness but personal contact with a counsellor did enhance effectiveness.	Critically low
	Hou et al (2013) [108]	7 (38)	Narrative synthesis	Internet	Body fat, weight, and dietary fat intake	0-12 months	In 71% (5/7) of studies, intervention groups lost more body fat, body weight, and dietary fat intake and maintained higher weight loss at 12 months.	Critically low
	Manzoni et al (2011) [62]	26 (26)	Narrative synthesis of all study types	Internet	Weight loss and weight loss maintenance	3-24 months	Internet-based weight loss interventions enhanced by professional feedback provided through the internet are more effective for weight loss than website-only programs but less effective than telephone counselling. 93% (13/14) of studies showed a further improvement in mean weight loss (weight maintenance) after the end of the trials.	Critically low
	Seo et al (2015) [69]	31 (31)	Meta-analysis of RCTs	Internet	Waist circumference	4 weeks-2 years	Internet-based interventions showed a significant reduction in waist circumference (mean change –2.99 cm; 95% CI −3.68 to −2.30; I^2^=93.3%) and significantly better effects on waist circumference loss (mean loss 2.38 cm; 95% CI 1.61 to 3.25; I^2^=97.2%) than minimal interventions such as information-only groups; no differences with respect to waist circumference change between internet-based interventions and paper-, phone-, or person-based interventions (mean change −0.61 cm; 95% CI −2.05 to 0.83; *P*=.42; k=31).	Critically low
	Sherrington et al (2016) [70]	12 (12)	Meta-analysis of RCTs	Internet	Weight loss	3-24 months	The internet-delivered weight loss interventions providing personalized feedback resulted in an MD of 2.13 kg (*P*<.001) greater weight loss in comparison with control groups receiving no personalized feedback. Heterogeneity levels showed considerable and significant heterogeneity (I^2^=99%; *P*<.001) between control groups not receiving personalized feedback and the internet-delivered weight loss interventions providing personalized feedback.	Critically low
	Elaheebocus et al (2018) [56]	11 (134)	Narrative synthesis of RCTs	Social media	Body weight	6-48 months	82% (9/11) of studies using web-based social networks had positive results for weight loss.	Critically low
	Maher et al (2014) [61]	5 (10)	Narrative synthesis	Social media	Weight	8 weeks-24 months	Findings were mixed, from negligible to large effect sizes for weight loss.	Critically low
	Mita et al (2016) [65]	10 (16)	Meta-analysis of RCTs	Social media	Weight change	1-12 months	Meta-analysis of all trials showed no significant differences for body weight (significant mean difference 0.07; 95% CI −0.17 to 0.20).	Moderate
	Williams et al (2012) [75]	10 (22)	Meta-analysis of RCTs	Social media	BMI; body weight; diet	3 months-24 months	Meta-analysis showed no significant differences in changes in weight (SMD 0; 95% CI −0.19 to 0.19; 10 studies); however, pooled results from 5 studies showed a significant decrease in dietary fat consumption with social media (SMD −0.35; 95% CI −0.68 to −0.02).	Critically low
	Willis et al (2017) [76]	5 (5)	Narrative synthesis of all study types	Social media	Body weight; body composition; blood pressure; and blood markers	8 weeks-6 months	100% (5/5) of studies reported a reduction in baseline weight. 60% (3/5) of studies reported significant decreases in body weight when online social networks was paired with health educator support. Only one study reported a clinically significant weight loss of 55%.	Critically low
	Bacigalupo et al (2013) [50]	5 (7)	Narrative analysis of RCTs	Mobile	Weight loss and BMI	9-52 weeks	Strong evidence for weight loss in the short term with moderate evidence for the medium term.	Low
	Covolo et al (2017) [104]	21 (40)	Narrative synthesis of RCTs	Mobile	BMI and waist circumference	6-12 months	62% (13/21) of studies did not find a statistical difference in changes in weight. 24% (5/21) of studies found that a mobile app was more effective compared with controls (*P*<.05). In 3 studies, this did not differ significantly between the 2 groups.	Critically low
	Head et al (2013) [107]	3 (19)	Meta-analysis of RCTs	Mobile (SMS text messaging)	Weight	Mean 81.26 days	The weighted mean effect size for weight loss was Cohen *d*=0.255 (95% CI .056 to .455; *P*=.01; k=3).	Critically low
	Liu et al (2015) [59]	9 (14)	Meta-analysis of RCTs	Mobile	Weight and BMI	3-30 months	Compared with the control group, mobile phone intervention was associated with significant changes in body weight and body mass index (weight [kg]/height (m^2^) of −1.44 kg (95% CI −2.12 to −0.76) and −0.24 units (95% CI −0.40 to −0.08), respectively; no differences between shorter and longer trials (< or ≥6 months; k=22).	Critically low
	Lyzwinksi et al (2014) [60]	8 (17)	Meta-analysis of RCTs	Mobile	Body weight and BMI	8 weeks-12 months	75% (6/8) of studies of mobile phone interventions found significant changes in weight favoring the mobile phone intervention groups over the controls; the meta-analysis generated a medium, significant effect size of 0.430 (95% CI 0.252 to 0.609; *P*≤.01), favoring mobile interventions.	Critically low
	Palmer et al (2018) [111]	3 (71)	Meta-analysis of RCTs	Mobile	Body weight and triglyceride levels	24 hours-6 months	There were, at best, modest benefits of diet and physical activity interventions. The effect of SMS text messaging–based diet and physical activity interventions on incidence of diabetes was pooled (risk ratio 0.67; 95% CI 0.49 to 0.90; I^2^=0.0%); end point weight was pooled (MD −0.99 kg; 95% CI −3.63 to 1.64; I^2^=29.4%); percentage change in weight was pooled (MD −3.1; 95% CI −4.86 to −1.3; I^2^=0.3%); and triglyceride levels was pooled (MD −0.19 mmol/L; 95% CI −0.29 to −0.08; I^2^=0%).	Moderate
	Schoeppe et al (2016) [68]	10 (27)	Narrative synthesis of RCTs, randomized trials, controlled trials, and pre- and poststudies	Mobile	Physical activity; diet; weight status; BMI; blood pressure; sedentary behavior; and fitness	1-24 weeks	40% (4/10) of studies that measured weight reported significant improvement in weight status; apps were more successful when used alongside other intervention components than when used alone.	Critically low
	Siopis et al (2015) [71]	6 (14)	Meta-analysis of RCTs, quasi-RCTs, and pre-post studies	Mobile	Body weight and BMI	8 weeks-12 months	The weighted mean change in body weight in intervention participants was −2.56 kg (95% CI −3.46 to −1.65) and in controls, −0.37 kg (95% CI −1.22 to 0.48).	Critically low
	Wieland et al (2012) [74]	18 (18)	Meta-analysis of RCTs, quasi-RCTs, and quasiexperimental studies	Computer based	Weight	4 weeks-30 months	At 6 months, computer-based interventions led to greater weight loss than minimal interventions (MD −1.5 kg; 95% CI −2.1 to −0.9; 2 trials) but less weight loss than in-person treatment (MD 2.1 kg; 95% CI 0.8 to 3.4; 1 trial). At 6 months, computer-based interventions were superior to a minimal control intervention in limiting weight regain (MD −0.7 kg; 95% CI −1.2 to −0.2; 2 trials) but not superior to infrequent in-person treatment (MD 0.5 kg; 95% −0.5 to 1.6; 2 trials).	Critically low
	Afshin et al (2016) [99]	35 (224)	Narrative synthesis and meta-synthesis for RCTs and quasiexperimental studies	Various: internet and mobile	Weight	3-30 months	69% (24/35) of studies reported significant improvements in adiposity following the intervention. 81% (13/16) of RCTs reported significant reductions in adiposity; using the internet in the weight loss program resulted in 0.68 kg (95% CI 0.08 to 1.29 kg) additional weight reduction over a period of 3 to 30 months; in studies finding significant weight reduction, the magnitude of weight change ranged from 1 to 6 kg after 6 months of follow-up.	Critically low
	Allen et al (2014) [48]	38 (39)	Narrative synthesis of randomized trials	Various: internet, messaging, chat rooms, and mobile	Weight loss	5 weeks-24 months	53% (21/39) of RCTs reported statistically significant weight loss in the intervention group as compared with the control group; the proportion varied by mode of delivery, the highest proportion of successful trials involving SMS text messaging or email (67%), followed by online chat rooms (50%), web-based (48%), and self-monitoring with technology (43%).	Critically low
	Bassi et al (2014) [51]	8 (28)	Narrative analysis of RCTs	Various: internet and mobile	BMI and weight	12 months	Results were mixed; 2 studies reported significant improvements with weight loss; however, effects were typically short lived, and more weight is regained in a primarily technology-based approach, as compared with personal contact.	Critically low
	Carvalho de Menzes et al (2016) [53]	18 (18)	Narrative synthesis of all study types	Various: email, telephone, face-to-face, and websites	Fat consumption, fruit and vegetable consumption, and physical activity	1-36 months	Approximately half the studies showed weight loss in the intervention group.	Critically low
	Coons et al (2012) [54]	13 (13)	Narrative synthesis of RCTs	Various: PDA^f^, web-based, and wearables	Body mass; BMI; BP^g^; waist circumference; RHR^h^; physical activity; body fat percentage; energy intake; and EE^i^	12 weeks-24 months	50% (6/12) of weight loss trials reported significantly greater weight loss among individuals randomized to technology interventions compared with controls; insufficient evidence to determine the effectiveness of interventions for weight maintenance.	Critically low
	Dutton et al (2014) [55]	18 (22)	Narrative synthesis of all study types	Various: mobile, internet, and podcasts	Weight	3 weeks-24 months	67% (12/18) of trials found significant differences in weight loss at one or more assessments.	Critically low
	Maxwell (2015) [64]	Not reported	Narrative synthesis of all study types	Technology interventions, including web-based and mobile	Healthy eating and active living	Not reported	Men participate in technology-based healthy lifestyle interventions less than women; maintenance of behavior is challenging.	Critically low
	Podina and Fodor (2018) [66]	43 (47)	Meta-analysis of RCTs	Various: mobile messaging, mobile app, and website	Weight, BMI, waist circumference, and percentage of body fat	3-24 months	Standard active treatment was more effective than eHealth interventions with regard to weight (*g*=−0.31; 95% CI − 0.43 to −0.20). There was a statistically significant, albeit small effect size favoring eHealth interventions relative to passive control groups for weight (*g*=0.34; 95% CI 0.24 to 0.44) and behavioral outcomes (*g*=0.17; 95% CI 0.07 to 0.27).	Critically low
	Ryan et al (2019) [67]	6 (6)	Narrative synthesis of randomized trials	Various: mobile or internet	Weight loss	5 weeks-24 months	Tailored interventions were found to be more effective in supporting weight loss than generic or waitlist controls in 66% (4/6) of articles. Effect sizes were very small to moderate, with evidence of fluctuations in effect sizes and differences of effect between tailored and nontailored interventions, and between tailoring types, over time.	Critically low
**Nonactive controls**								
	An et al (2017) [49]	21 (22)	Meta-analysis of RCTs, pre-post studies, and cohort studies	Social media	Physical activity; sedentary behavior; diet; BMI; hip-waist ratio; body fat; and waist circumference	2-102 weeks	Social media–based interventions were found to reduce body weight by 1.01 kg (95% CI 0.45 to 1.57), BMI by 0.92 kg/m^2^ (95% CI 0.29 to 1.54), and waist circumference by 2.65 cm (95% CI 0.86 to 4.43).	Critically low
	Tang et al (2016) [72]	18 (27)	Meta-analysis of RCTs	Various	Body weight; BMI; and waist circumference	1-24 months	Participants receiving internet-based, self-directed interventions lost significantly more weight than those receiving minimal intervention or no treatment (MD −1.72 kg; 95% CI −2.60 to −0.84; significant mean difference −0.45; 95% CI −0.67 to −0.23; I^2^=80%; *P*<.001) and a significantly greater reduction in BMI levels than those receiving no treatment or minimal intervention (MD −0.47 kg/m^2^; 95% CI −0.81 to −0.14; significant mean difference −0.32; 95% CI −0.61 to −0.03; I^2^=90%; *P*=.03; 13 evaluations). There was a greater reduction in BMI (MD 0.54 kg/m^2^) and waist circumference (2.81 cm) at 0-4 months follow-up than at later times (k=27).	Critically low
**Active controls**								
	Beleigoli et al (2019) [52]	11 (11)	Meta-analysis of RCTs	Internet	Weight and BMI	3-12 months	Compared with offline interventions, digital interventions led to a greater short-term (<6 months follow-up) weight loss (MD −2.13 kg; 95% CI −2.71 to −1.55; 393 participants; high-certainty evidence) but not in the long-term (MD −0.17 kg; 95% CI −2.10 to 1.76; 1104 participants; moderate-certainty evidence).	Critically low
	Kodama et al (2012) [58]	23 (23)	Meta-analysis of RCTs	Internet	Weight loss	3-30 months	Using the internet had a modest but significant additional weight loss effect compared with nonweb user control groups (−0.68 kg; *P*=.03). Internet-based interventions were effective for weight loss (−1.00 kg; *P*<.001) but not a substitute for face-to-face support (+1.27 kg; *P*=.01). An additional effect on weight control was observed when the aim of using the internet was initial weight loss (−1.01 kg; *P*=.03) but was not observed when the aim was weight maintenance (+0.68 kg; *P*=.26); furthermore, it was effective to use the internet as an adjunct to face-to-face care (−1.00 kg; *P*<.001) but adverse effects on weight loss were found when it was used as a substitute (+1.27 kg; *P*=.01). The weight loss effect was insignificant (−0.20 kg; *P*=.75) in studies with educational periods ≥12 months and was significant in studies with an educational period <6 months (−1.55 kg; *P*=.001).	Critically Low

^a^RCT: randomized controlled trial.

^b^HbA_1c_: glycated hemoglobin.

^c^LDL: low-density lipoprotein.

^d^MD: mean difference.

^e^SMD: standardized mean difference.

^f^PDA: personal digital assistant.

^g^BP: blood pressure.

^h^RHR: resting heart rate.

^i^EE: energy expenditure.

Internet interventions were found to be somewhat effective. A total of 5 narrative syntheses found them to be effective in approximately half of the studies [47,62,99,100,108]. In total, 3 meta-analyses quantified weight loss and other health effects. A total of 3 reviews found a significant reduction in weight: mean difference –1.34 kg [102], SMD 2.13 kg [70], and a 0.68-kg additional weight reduction over a period of 3 to 30 months [99]. A fourth review found that internet interventions significantly reduced waist circumference (mean change −2.99 cm) [69]. However, stratified analysis suggested that internet interventions were effective when used in combination with in-person counseling (−1.93 kg), rather than as a substitute [98]. This finding was supported by a narrative synthesis, which reported that internet interventions were more effective when they were enhanced to offer more than just educational resources (several studies found medium effect sizes) [62].

There were mixed results regarding the effectiveness of social media interventions. A total of 2 reviews were favorable [49,56], including a meta-analysis that found that social media–based interventions reduced body weight by 1.01 kg, BMI by 0.92 kg/m^2^, and waist circumference by 2.65 cm but did not find significant changes in body fat or body fat percentage [49]. A total of 2 narrative syntheses found effects that were small and not meaningful [61,76]. Two meta-analyses found no significant effects on the diverse primary outcomes of the studies or on body weight [65,75].

Most reviews of mobile phone interventions found that they were effective in achieving weight loss via diet and physical activity. One meta-analysis found a statistically significant medium effect size (Cohen *d*=0.430) in favor of mobile phone interventions [60]. Two others quantified the change in terms of body weight: mobile phone interventions were associated with significant changes in body weight of −1.44 kg and in BMI of −0.24 kg/m^2^ compared with controls [59]; the weighted mean body weight change in intervention participants was −2.56 kg compared with −0.37 kg in controls [71]. These results were supported by 3 narrative syntheses, which found evidence that mobile interventions led to weight loss [50,68,99]. However, one review of apps to promote healthy lifestyles found that most trials did not show significant differences [104], and one of the reviews that looked at the use of apps for diet, physical activity, and sedentary behavior, which concluded that apps led to weight loss, also concluded that the apps were more successful when used alongside other intervention components than when used alone [68].

In total, 4 of 5 narrative syntheses that ranged over a variety of digital interventions found that around half of the interventions were effective compared with controls [48,51,53,54], with only one finding that a large majority (81%) reported a significant reduction in adiposity [99]. One of these studies found that most trials reported within-group weight loss, even when there was no difference between digital interventions and controls [54]. Another study compared the success of different modes of delivery, finding the highest proportion of successful trials involving SMS text messaging or email (67%), followed by online chat rooms (50%), web-based (48%), and self-monitoring with technology (43%) [48].

##### Effectiveness of Digital Interventions on Diet and Physical Activity (Weight Loss) Compared With Nonactive Intervention Controls

A total of 6 reviews that reported results for digital interventions for diet and physical activity to no intervention or minimal intervention controls all agreed that digital interventions were more effective [66,67,69,70,72,74]. Table 5 provides a summary of the results.

Internet interventions were more effective than nonactive controls according to 3 meta-analyses and 1 narrative synthesis [67,69,70,72]. Internet interventions led to a greater reduction in waist circumference (mean change –2.99 cm, 95% CI −3.68 to −2.30, I2=93.3% vs 2.38 cm, 95% CI 1.61 to 3.25, I2=97.2%) [69]; internet-delivered personal feedback led to greater weight loss (mean difference 2.14 kg) [70]; and self-directed internet interventions led to significantly more weight loss (mean difference −1.56 kg) and showed a significantly greater reduction in BMI (mean difference −0.41 kg/m^2^) [72].

Similar effects were observed for computer-based interventions, which led to greater weight loss at 6 months (mean difference −1.5 kg) and were superior to limiting weight regain (mean difference −0.7 kg) [74].

A narrative review of tailored internet interventions found that effect sizes ranged from very small to moderate [67]. This was supported by a meta-analysis that covered various digital interventions, which found small effect sizes favoring digital interventions for weight (*g*=0.34) and behavioral outcomes (*g*=0.17) [66].

##### Effectiveness of Digital Interventions on Diet and Physical Activity (Weight Loss) Compared With Active Controls

The effects of digital interventions on diet and physical activity (with regard to weight loss) compared with active controls were mixed. A total of 2 meta-analyses found no differences between web-based interventions and active offline interventions for weight loss outcomes [52,69]. One meta-analysis of a range of interventions found that standard active treatment was more effective for weight loss (*g*=0.31) [66]. However, one meta-analysis found that using the internet had a modest but significant additional weight loss effect compared with offline control groups (−0.68 kg; *P*=.03), with a subgroup analysis showing that the internet was effective compared with controls for achieving weight loss (weight change=−1.01 kg) but not weight maintenance [58]. The same meta-analysis also found that it was effective to use the internet as an adjunct to face-to-face care (−1 kg; *P*<.001) but that adverse effects on weight loss were found when it was used as a substitute (+1.27 kg; *P*=.01) [58].

Computer-based interventions led to less weight loss than in-person treatment (mean difference 2.1 kg) and were not superior to infrequent in-person treatment in limiting weight regain at 6 months [74].

##### Sustainability of Effects on Diet and Physical Activity (Weight Loss) at Follow-Up

Although technology-related health interventions may be effective, the maintenance of behavior is challenging [64], and there is insufficient evidence to determine the effectiveness of digital interventions on weight maintenance [54]. One review of internet interventions reported that, in studies finding significant weight reduction, the magnitude of weight change ranged from 1 to 6 kg after 6 months of follow-up [99].

Many studies included in the reviews had short follow-ups. Where longer follow-ups were reported, effectiveness typically diminished over time. Examples of the diminishing effects are clear in the 2 reviews. One meta-analysis of internet-delivered personal feedback found a greater reduction in BMI (mean difference 0.54 kg/m^2^) and waist circumference (2.81 cm) at 0 to 4 months follow-up than at later times compared with undefined control groups [72]. A narrative synthesis supports these findings, concluding from multiple high-quality randomized controlled trials (RCTs) that weight loss occurs for a short term through mobile interventions, with moderate evidence for the medium term [50]. Two other meta-analyses reported significantly greater weight loss in favor of digital interventions in the medium term only (<6 months): 2.13 kg [52] and 1.55 kg [58]. One meta-analysis assessed differences between trials of different lengths, reporting no differences between shorter and longer trials (<6 or ≥6 months) [59].

#### Smoking

##### Review Characteristics

There were 28 reviews on smoking (see Multimedia Appendix 6 for summary characteristics).

##### Effectiveness of Digital Interventions on Smoking Compared With Mixed (Active and Nonactive) Controls

Digital interventions were generally effective across different modes of delivery (Table 6).

**Table 6:** Results of reviews on smoking, ordered by type of control and further ordered by mode of delivery of intervention.

Review		Relevant studies, n (total studies)	Method of synthesis	Interventions	Outcomes	Follow-up	Summary of findings	AMSTAR-2 rating
**Mixed (active and nonactive) controls**								
	Afshin et al (2016) [99]	22 (224)	Narrative synthesis of RCTs^a^ and quasiexperimental studies	Internet and mobile interventions	Abstinence	1 week-2 years	77% (17/22) of studies reported a significant increase in abstinence. In studies reporting benefits, the OR^b^ for 7-day abstinence at 6 months ranged from 1.6 (95% CI 1.1 to 2.4) to 2.7 (95% CI 1.8 to 4.0).	Critically low
	Boland et al (2016) [83] (Smoking only)	13 (13)	Meta-analysis of RCTs	Various: website or computer program and SMS text messaging	Abstinence	1 week-18 months	Interventions increased the odds of smoking cessation for disadvantaged groups at 1 month (OR 1.70; 95% CI 1.10 to 2.63), 3 months (OR 1.30; 95% CI 1.07 to 1.59), 6 months (OR 1.29, 95% CI 1.03 to 1.62) and 18 months postintervention (OR 1.83, 95% CI 1.11 to 3.01).	Low
	Aneni et al (2014) [100]	3 (29)	Narrative synthesis of RCTs	Internet	Cessation	12 months	3 follow-up studies that measured smoking cessation showed significant intervention effects, although they were assessed to be of low quality.	Critically low
	Chebli et al (2016) [103]	9 (16)	Narrative synthesis of RCTs	Internet	Cessation and reduction	1-12 months	Internet-based interventions may have a positive effect on smoking cessation. Several studies found that web-based use and number of log-ins was positively associated with quit outcomes.	Critically low
	Cheung et al (2017) [85] (Smoking only)	6 (45)	Narrative synthesis and meta-analysis of RCTs and quasi-RCTs	Internet	Cessation	>4 weeks	Only 13% (6/45) of studies provided data on effectiveness, with 66% (4/6) of studies demonstrating effectiveness. Smokers using a web-based cessation intervention were 1.15 to 2.84 times more likely to become a former smoker compared with the control condition (with a pooled RR^c^ 1.39; 95% CI 1.18 to 1.65).	Critically low
	Gainsbury and Blaszczynski (2010) [87] (Smoking only)	7 (9)	Narrative synthesis of RCTs and pre-experimental studies	Internet	Abstinence, tobacco use, smoking status, compliance, nicotine dependence, carbon monoxide markers, and toxicity	3-12 months	86% (6/7) of studies reported significantly greater self-reported smoking quit rates or abstinence at the end of the treatment trial for participants in the internet intervention compared with controls. Several trials found improvements at 3, 6, and 12 months.	Critically low
	Graham et al (2016) [88] (Smoking only)	40 (40)	Meta-analysis of RCTs	Internet	Abstinence	7 days-3 months	Pooled results from 15 trials (24 comparisons) found a significant effect in favor of experimental internet interventions (RR 1.16; 95% CI 1.03 to 1.31; I^2^=76.7%).	Critically low
	Hutton et al (2011) [90] (Smoking only)	15 (21)	Narrative synthesis of RCTs	Internet	Cessation	>1 month	Two RCTs found that a multicomponent intervention with web and nonweb-based elements was more efficacious than a self-help manual, and one of the 2 RCTs found that web-based interventions may be more effective than no treatment. Three trials provided insufficient evidence to demonstrate whether web-based interventions were more efficacious than counselling. Tailored websites in 2 RCTs and greater website exposure in 86% (6/7) of RCTs were associated with higher rates of abstinence.	Critically low
	Lustria et al (2013) [110]	8 (40)	Meta-analysis of experimental and quasiexperimental studies	Internet	Abstinence	30 days-6 months	Web‐based, tailored interventions had significantly greater improvement in smoking outcomes compared with control conditions, with small effects, Cohen *d*=0.151 (k=8; 95% CI 0.11 to 0.19; *P*<.001).	Critically low
	McCrabb et al (2019) [91] (Smoking only)	45 (45)	Meta-analysis of RCTs	Internet	Abstinence	1-18 months	Interventions were effective in the short term (OR 1.29, 95% CI 1.12 to 1.50; *P*=.001) and long term (OR 1.1.9, 95% CI 1.06 to 1.35; *P*=.004).	Low
	Shahab and McEwen (2009) [95] (Smoking only)	10 (11)	Meta-analysis of RCTs	Internet	Cessation	>1 month	Interactive interventions were effective compared with untailored booklets or emails (RR 1.8; 95% CI 1.4 to 2.3) increasing 6-month abstinence by 17% (95% CI 12 to 21%); no evidence was found of a difference between interactive and static interventions.	Critically low
	Taylor et al (2017) [97] (Smoking only)	61 (67)	Meta-analysis of RCTs and quasi-RCTs	Internet	Cessation	6-12 months	Interactive and tailored internet-based interventions with or without additional behavioral support are moderately more effective than nonactive controls at 6 months or longer, but there was no evidence that these interventions were better than other active smoking treatments.	Moderate
	Webb et al (2010) [113]	12 (85)	Meta-analysis of RCTs	Internet	Smoking abstinence	12 months	Interventions that targeted smoking abstinence tended to have small effects on behavior that did not reach statistical significance (Cohen *d*+=0.07; k=12; 95% CI −0.04 to 0.18).	Critically low
	Head et al (2013) [107]	5 (19)	Meta-analysis of RCTs	SMS text messaging	Smoking cessation	Mean 81.26 days	The weighted mean effect size for smoking cessation, Cohen *d*=0.447 (95% CI .367 to .526; *P*=.001; k=5).	Critically low
	Scott-Sheldon et al (2016) [94] (smoking only)	18 (20)	Meta-analysis of RCTs	SMS text messaging	Abstinence, cigarette use, quit attempts, and nicotine dependence	Not reported	SMS text messaging was associated with significantly greater odds of abstinence compared with controls: 7-day point prevalence (OR 1.38, 95% CI 1.22 to 1.55; k=16) and continuous abstinence (OR 1.63, 95% CI 1.19 to 2.24; k=7); interventions were also more successful in reducing cigarette consumption (Cohen *d*_+_=0.14; 95% CI 0.05 to 0.23; k=9).	Critically low
	Spohr et al (2015) [96] (smoking only)	10 (13)	Meta-analysis of RCTs	SMS text messaging	Cessation	3 and 6 months	Interventions generally increased quit rates compared with controls (OR 1.36, 95% CI 1.23 to 1.51). Intervention efficacy was higher in studies with a 3-month follow-up compared with a 6-month follow-up.	Critically low
	Palmer et al (2018) [111]	18 (71)	Meta-analysis of RCTs	Mobile	Abstinence and cessation (verified biochemically)	24 hours-6 months	The effect of SMS text messaging–based smoking cessation support on biochemically verified continuous abstinence was pooled relative risk, RR 2.19 (95% CI 1.80 to 2.68; I^2^=0%) and on verified 7-day point prevalence of smoking cessation was pooled RR 1.51 (95% CI 1.06 to 2.15; I^2^=0%).	Moderate
	Whittaker et al (2016) [98] (smoking only)	12 (12)	Meta-analysis of RCTs and quasi-RCTs	Mobile	Cessation	6 months	Smokers who received support programs were 1.7 times more likely to stay quit than smokers who did not receive the programs (9.3% quit with programs compared with 5.6% who quit with no programs). Most of the studies were of programs relying mainly on text messages.	Moderate
	Danielsson et al (2014) [86] (smoking only)	21 (74)	Narrative synthesis of RCTs	Various	Abstinence	>3 months	The studies showed mixed results regarding internet interventions and smoking, with some positive effects for the smoking cessation program that combined the use of both the internet, mobile phones (SMS text messaging), and email.	Critically low
	HIQA (2017) [89] (smoking only)	12 (143)^d^	Network meta-analyses of RCTs	Various: internet and mobile	Cessation	6-12 months	Internet-based interventions are superior to control (brief advice or written materials; RR 1.43, 95% CI 1.02 to 2.00; *P*=.04; k=5); Internet-based interventions are superior to doing nothing (RR 1.46, 95% CI 1.18 to 1.81; *P*<.001; k=3); mobile phone–based interventions appear to have similar effectiveness to control (RR 1.18, 95% CI 0.88 to 1.60; *P*=.27; k=3); no evidence of difference between mobile phone–based interventions and internet (RR 1.43, 95% CI 0.88 to 2.31; *P*=.15; k=1).	Low
	Hou et al (2013) [108]	5 (38)	Narrative synthesis of studies with comparison or control groups	Web-based computer programs	Cessation	Not reported	2 studies found higher cessation rates in intervention groups than control. 3 studies found no significant differences in quit rates at the end of the intervention or at follow-ups.	Critically low
	Myung et al (2009) [92] (smoking only)	22 (22)	Meta-analysis of RCTs	Various: internet or computer based	Abstinence and biochemical markers	>3 months	Intervention groups had a significant effect on smoking cessation (RR 1.44; 95% CI 1.27 to 1.64). Similar findings were observed in web-based interventions (RR 1.40; 95% CI 1.13 to 1.72) and in computer-based interventions (RR 1.48; 95% CI 1.25 to 1.76).	Critically low
	Naslund et al (2017) [93] (smoking only)	7 (7)	Narrative synthesis of all study types	Social media	Cessation	30-365 days	71% (5/7) of studies reported significant effects on smoking-related outcomes such as greater abstinence, reduction in relapse, and an increase in quit attempts.	Critically low
	Elaheebocus et al (2018) [56]	7 (134)	Narrative synthesis of RCTs	Social media	Cessation	6-48 months	100% (7/7) of studies on smoking cessation using web-based social networks had positive results.	Critically low
	Rooke et al (2010) [112]	13 (34)	Meta-analysis of RCTs	Computer based	Abstinence and reduction	1-156 weeks	The weighted average effect size (Cohen *d*) was 0.14; *P*<.001 for studies addressing tobacco use.	Critically low
**Nonactive controls**								
	Chen et al (2012) [84] (smoking only)	60 (60)	Meta-analysis of RCTs and quasi-RCTs	Various: computer and other electronic aids	Cessation	2 days-30 months	Computer and other electronic aids increase the likelihood of cessation compared with no intervention or generic self-help materials but the effect is small (prolonged abstinence: RR 1.32; 95% CI 1.21 to 1.45).	Low
	Krebs et al (2010) [109]	32 (88)	Meta-analysis of RCTs	Computer tailored	Abstinence	24 hours-9 months	Mean effect for the 32 studies reporting point prevalence outcome was *g*=0.16 (95% CI 0.12 to 0.19); mean effect for the 16 studies reporting prolonged abstinence measures was *g*=0.24 (95% CI 0.20 to 0.31).	Low
**Active controls**								
	Covolo et al (2017) [104]	2 (40)	Narrative synthesis of RCTs	Mobile apps	30-day point prevalence cessation	30 days	1 trial compared 2 apps and found no evidence of any difference; the other found text messaging produced more abstinence than an app (*P*<.05).	Critically low

^a^RCT: randomized controlled trial.

^b^OR: odds ratio.

^c^RR: risk ratio.

^d^In total, 12 relevant studies were included in the meta-analysis.

Internet interventions were generally found to be effective (Table 6). A total of 3 relevant meta-analyses found small or small-to-medium effects [85,89,91]: pooled RR 1.39 [85]; RR 1.40 [92], and RR 1.43 [88] and a short-term (≤6 months) OR 1.29 (95% CI 1.12 to 1.50; *P*=.001) [91]; the latter showed that internet interventions were also successful for the individual outcomes of *prolonged abstinence*, that is, not smoking since a quit date (OR 1.43) and *30-day point prevalence abstinence*, that is, not smoking one or more days before the follow-up (OR 1.75) [91]. However, one narrative synthesis noted that although the 3 studies that measured smoking cessation all showed significant intervention effects, they were all assessed to be of low quality [100].

The 3 reviews of mobile interventions that focused on SMS text messaging found it to be effective, again with small effects: Cohen *d*=0.14 [94]; OR 1.36 [96]; and OR 1.36 [98]. A fourth meta-analysis differentiated between specific outcomes and found a medium effect (RR 2.19) on biochemically verified continuous abstinence, and a small-to-medium effect (RR 1.51) on verified 7-day point prevalence of smoking cessation was pooled [111]. However, 2 reviews (including 1 meta-analysis) with information on mobile interventions in general did not find that they were effective [89,99].

A meta-analysis of computer-delivered interventions also found a small but significant effect size associated with studies addressing tobacco use (Cohen *d*=0.14) [112].

There is mixed evidence from reviews that cover a variety of digital technologies. One narrative synthesis [99] and one meta-analysis found that they were effective, with interventions increasing the odds of smoking cessation at 1 month (OR 1.70) [83]. However, another narrative synthesis that included both phone and internet interventions found that there were mixed results [86].

A total of 2 reviews of social media interventions with narrative syntheses both found favorable effects of the interventions on outcomes related to smoking cessation [56,93].

As seen above, separate reviews of internet, mobile, and computer-delivered interventions found small effects for each. One meta-analysis looked for differences in effect sizes between different modes of intervention and found no statistically significant evidence of any differences in effect sizes of internet interventions, intensive advice, telephone support, individual counseling, or group behavior therapy [89].

##### Effectiveness of Digital Interventions on Smoking Compared With Nonactive Controls

There were small effects of digital interventions compared with nonactive controls for all modes of delivery (Table 6). Meta-analyses found small effects for pooled modes of digital interventions compared with controls: 1 month OR 1.70 (k=13) [83]; pooled estimate for prolonged abstinence, RR 1.32 (k=60) [84], and pooled estimate for point prevalence abstinence, RR 1.14 [84]. Internet interventions were more effective than nonactive controls [88-90,92,97], with 3 meta-analyses finding small effects compared with nonactive controls: RR 1.15 [97], RR 1.46 [89], and RR 1.60 [88]. Computer-tailored interventions had effects sizes of *g*=0.16 for point prevalence outcomes and *g*=0.24 for prolonged abstinence [109]. Mobile phone–based interventions appeared to have similar effectiveness to minimal controls (RR 1.18) and were more effective than no intervention (typically either waiting list control or no further contact until follow-up) [89].

##### Effectiveness of Digital Interventions on Smoking Compared With Active Controls

Most reviews concluded that internet interventions were not more effective than active controls, with 3 reviews (including 2 meta-analyses) not finding differences [88,90,97]. This included a meta-analysis that found no significant effects of internet interventions compared with face-to-face counseling or telephone counseling [88]. However, one narrative synthesis was more positive about the greater effect of internet interventions compared with conventional ones [99].

There was no information on mobile interventions compared with active controls, but one meta-analysis with mixed controls noted that the summary effect sizes favored the treatment groups even when 18 of the 20 (90%) controlled trials used an active control and 12 (66%) of these active controls included some smoking-related content, including smoke-free websites, self-help guidebooks, and smartphone apps [94].

##### Sustainability of Effects on Smoking at Follow-Up

There was agreement that the effects of internet interventions were sustained for up to 6 months [83,87,91,95]. This was quantified as interventions increasing the 6-month abstinence by 17% [95], increasing the odds of cessation at the 6-month follow-up (OR 1.29) [83], and increasing the likelihood of *prolonged abstinence* (ie, not smoking since a quit date; OR 1.19) [91]. However, there was disagreement about whether there were significant effects at 12-month follow-ups, with 2 reviews in favor [87,89], one quantifying the quit rate at 12 months as 8% [89] but another reporting that the positive results of internet interventions were not generally maintained at 1-year follow-up assessments [103].

There is mixed evidence on the sustainability of the effects of mobile interventions. A total of 2 meta-analyses found that mobile interventions were effective in the medium term, with a quit rate of 13% [89] and effects on biochemical measures of quitting at 6 months (RR 1.83) [98]. However, a study of SMS text messages suggested that any effect was not sustained [96].

Regarding computer-delivered interventions, one meta-analysis found that computer programs increased the odds of cessation at the 3-month (OR 2.04), 12-month (OR 1.68), and 18-month follow-up (OR 1.83) [83]. A meta-analysis of computer-delivered interventions did not find evidence of differential effects depending on the length of follow-up or number of sessions [112].

A total of 2 reviews that considered a range of different technologies found evidence of sustained effects. A narrative synthesis of internet and mobile interventions found that the OR for 7-day abstinence at 6 months ranged from 1.6 to 2.7 [99]. A meta-analysis found that digital interventions increased the odds of smoking cessation at 3 months (OR 1.30), 6 months (OR 1.29), and 18 months (OR 1.83) postintervention [83].

In a pooled analysis of web-based and computer-based interventions, the smoking cessation rate was 14.8% in the intervention group and 14.3% in the control group at the short-term 3-month follow-up (*P*=.42), 11.7% and 7.0% at the midterm 6- to 10-month follow-up ( *P*<.001), and 9.9% and 5.7% at the long-term 12-month follow-up (*P*<.001), respectively [92].

One meta-analysis that covered multiple intervention types found that SMS text messaging had the highest OR (2.81) at the 1-month follow-up, followed by the use of computer programs at the 3-month follow-up (OR 2.04), 12-month follow-up (OR 1.68), and 18-month follow-up (OR 1.83) and websites at 6 months (OR 1.37), while a DVD intervention and integrated videotelephony did not increase the odds of cessation compared with no intervention [83].

#### Alcohol

A total of 13 papers covered alcohol [73,77-82,99,103,104,111-113]. Multimedia Appendix 5 presents a breakdown of the study characteristics.

##### Effectiveness of Digital Interventions on Alcohol Consumption Compared With Mixed (Active and Nonactive) Controls

In total, 7 of 8 (88%) reviews with mixed controls reported favorable evidence that digital interventions were effective in decreasing alcohol consumption (Table 7), although one review noted that the controls were just as effective [103]. A total of 2 meta-analyses reported small effect sizes (Cohen *d*=0.26 [112] and Cohen *d*=0.14 [113]). A narrative synthesis of interventions using novel technologies found that, in studies finding benefits and reporting compliance with drinking recommendation as an outcome, the OR for drinking within the recommended limit ranged from 1.7 to 3.7 (small/medium to medium/large effects) [99]. In contrast, the sixth review, which surveyed mobile phone interventions, reported that the results were inconclusive for alcohol reduction, and the authors declined to perform a meta-analysis because the results were self-reported and therefore at risk of overstating the benefits [111].

**Table 7:** Effectiveness of digital interventions on alcohol consumption, sorted by controls and further ordered by intervention type.

Review			Relevant studies, n (total studies)		Method of synthesis		Interventions		Outcomes		Follow-up		Summary of findings		AMSTAR-2 rating
**Mixed (active and nonactive) controls**															
	Afshin et al (2016) [99]	47 (224)		Narrative synthesis of RCTs^a^ and quasiexperimental studies		Various: internet, mobile, computer software, and sensors		Alcohol frequency and quantity, binge drinking, estimated blood alcohol concentration, alcohol dependency, and Alcohol Use Disorder Identification Test scores		1 week-2 years		34% (39/47) of studies, 41 RCTs and 6 quasiexperimental, reported a significant decrease in alcohol use; 83% (33/41) of RCTs reported statistically significant benefits.		Critically low	
	Chebli et al (2016) [103]	2 (16)		Narrative synthesis of RCTs		Internet		Cessation and reduction of alcohol		1-12 months		Both studies demonstrated positive treatment outcomes in both arms, but there were no differences between internet intervention and control.		Critically low	
	Kaner et al (2017) [77] (Alcohol only)	57 (57)		Narrative synthesis and meta-analysis of RCTs		Computer and mobile		Alcohol consumption and frequency		1-12 months		Alcohol consumption reduced by approximately 23 g per week (95% CI 15 to 30) at follow-up (1-12 months; based on 41 studies). Frequency of consumption reduced (based on 15 studies): participants who engaged with digital interventions had less than one drinking day per month fewer than no intervention controls (moderate‐quality evidence); had about one binge drinking session less per month in the intervention group (moderate‐quality evidence); and drank one unit per occasion less than no intervention control participants (moderate‐quality evidence). Compared with face-to-face interventions, there was no difference in alcohol consumption at the end of follow-up (mean difference 0.52 g/week; 95% CI −24.59 to 25.63; low‐quality evidence).		Low	
	Kolar et al (2015) [78] (Alcohol only)	2 (18)		Narrative synthesis of all studies		Internet		Alcohol quantity and frequency		1 month		100% (2/2) of studies found reduced alcohol consumption in both arms but no significant differences between arms.		Critically low	
	Palmer et al (2018) [111]	8 (71)		Narrative synthesis of RCTs		Mobile		Self-report alcohol consumption		Not reported		The effects of alcohol reduction interventions were inconclusive.		Moderate	
	Rooke et al (2010) [112]	9 (34)		Meta-analysis of RCTs		Computer-delivered		Abstinence and reduction of alcohol		1-156 weeks		The weighted average effect size (Cohen *d*) was 0.20 (*P*<.001).		Critically low	
	Vernon et al (2010) [81] (Alcohol only)	15 (15)		Narrative synthesis of all studies		Computer-delivered		Alcohol consumption		30 days-12 months		All but one intervention showed significant improvement in at least one drinking-related outcome. However, interventions were heterogenous and preintervention alcohol consumption was not standardized.		Critically low	
	Webb et al (2010) [113]	9 (85)		Meta-analysis of RCTs		Internet		Alcohol consumption		Not reported		Small effects were observed for alcohol consumption (Cohen *d*_+_=0.14; k=9; 95% CI 0.00 to 0.27).		Critically low	
**Nonactive controls**															
	Black et al (2016) [82] (Alcohol only)	93 (93)		Meta-analysis of RCTs		Computer delivered		Alcohol consumption: total consumption over a period of time; average alcohol consumption per drinking occasion or drinking day; peak consumption—max consumed on one occasion. Frequency of heavy episodic drinking and of any alcohol consumption		Up to 2 years		Small effects averaging across timepoints, Cohen *d*=0.007 (heavy episodic drinking frequency) to Cohen *d*=0.15 (total consumption); in the short term, there were small-to-medium effects (Cohen *d*+=0.16 to 0.31) and significant effects on all outcomes except drinking frequency; in the medium-to-long term, they produced small (Cohen *d*+=0.07 to 0.12), significant effects on all outcomes.		Critically low	
	Covolo et al (2017) [104]	1 (40)		Narrative synthesis of RCTs		Mobile		Alcohol frequency		0-2 years		Contrary to expectation, it was found that the mobile app significantly increased the frequency of drinking occasions compared with the control group (*P*=.001).		Critically low	
	Riper et al (2011) [79] (Alcohol only)	9 (9)		Meta-analysis of RCTs		Internet		Alcohol consumption		Up to 12 months		An overall medium effect size (*g*=0.44; 95% CI 0.17 to 0.71; random effect model) in favor of the intervention groups was found.		Critically low	
	Riper et al (2014) [80] (Alcohol only)	16 (16)		Meta-analysis of RCTs		Internet		Alcohol consumption		1 to 12 months		A small but significant overall effect size in favor of internet interventions (*g*=0.20; 95% CI 0.13 to 0.27; *P*=.001) was found. Participants in internet-based interventions consumed approximately 22 g of ethanol less and were more likely to adhere to low-risk drinking guidelines (risk difference 0.13; 95% CI 0.09 to 0.17; *P*=.001).		Critically low	
	Tsoli et al (2018) [73]	4 (15)		Meta-analysis of RCTs		Interactive voice responses		Alcohol consumption		6 weeks-12 months		The meta-analysis of included studies demonstrated that interactive voice response–based interventions had no statistically significant effect on alcohol consumption (*g*=−0.077; 95% CI −0.162 to 0.007; k=4; *P*=.07).		Critically low	

^a^RCT: randomized controlled trial.

##### Effectiveness of Digital Interventions on Alcohol Consumption Compared With Nonactive Controls

In total, 4 of 6 (67%) reviews with nonactive controls found an effect of the intervention (Table 7). Of the 4 meta-analyses, 2 found a medium effect on alcohol consumption (*g*=0.44 [80] and *g*=0.44 [79]). A third found a small-to-medium effect on total alcohol consumption (Cohen *d*+=0.31), small effects on 2 other consumption measures, and a measure of frequency of heavy drinking episodes (Cohen *d*+=0.16-0.19) and no effect on drinking frequency [82]. The fourth review found no effect [73].

Two studies quantified the effect on alcohol consumption, finding a reduction in weekly consumption of 22 g of alcohol [80] and 23 g of alcohol [77], approximately 3 UK units. However, when studies with a high risk of bias were excluded, this number decreased to 11 g of alcohol (or 1.5 UK units) per week [77]. With regard to frequency of consumption, participants who were given digital interventions had less than one drinking day per month, which is fewer compared with no-intervention controls (moderate-quality evidence); had approximately 1 binge-drinking session less per month in the intervention group compared with no-intervention controls (moderate-quality evidence); and drank 1 unit per occasion, which is less than the no-intervention control participants (moderate-quality evidence) [77]. Participants in internet interventions were significantly more likely to adhere to low-risk drinking guidelines at the immediate posttreatment follow-up, compared with the no-intervention controls [80].

Only one review was negative. A narrative synthesis of mobile apps reported that there was a single RCT on alcohol reduction where, contrary to expectation, the frequency of drinking occasions was higher in the intervention group [104].

##### Effectiveness of Digital Interventions on Alcohol Consumption Compared With Active Controls

In total, 2 reviews that separated active controls found that there were no significant differences between the intervention and control groups [77,99], one of which was specifically compared with face-to-face controls (Table 7) [77].

##### Sustainability of Effects on Alcohol Consumption at Follow-Up

The effectiveness of the interventions seemed to decrease over time (Table 7). One meta-analysis, which reported small-to-medium effects in the short term (Cohen *d*+=0.16-0.19), found that in the medium to long term, there were small (Cohen *d*+=0.07-0.12), significant effects on all outcomes [82]. A total of 2 reviews reported that, for internet-based intervention studies where there were 3-, 6-, or 12-month follow-up data, no significant differences in effect remained in later follow-up [80,103]. In contrast, one review of computer- or mobile-delivered interventions found that positive differences in measures of drinking were seen at 1, 6, and 12 months [77], and one review of internet interventions found a medium effect size (*g*=0.39), lasting up to 6 or 9 months posttreatment, as compared with no intervention; the effects of the interventions beyond 9 months could not be assessed, but 2 studies in the review suggested that they had faded out by 12 months [79].

#### Other Combinations

##### Review Characteristics

A total of 11 reviews covered a number of areas of behavior [101-107,109,110,112,113]. The breakdown of their characteristics is shown in Multimedia Appendix 5.

##### Effectiveness of Other Digital Combination Interventions Compared With Mixed (Active and Nonactive) Controls

All 5 reviews of internet interventions concluded that they were effective in changing behavior (Table 8). A total of 3 meta-analyses found small effects: Cohen *d*=0.19 [105], Cohen *d*=0.139 [110], and Cohen *d*=0.16 [113]. However, one study reported that the effect sizes were heterogeneous [113]. The fourth meta-analysis quantified the effects on health outcomes, finding statistically significant reductions in systolic blood pressure (mean difference –2.66 mm Hg), diastolic blood pressure (mean difference –1.26 mm Hg), glycated hemoglobin level (mean difference –0.13%), and low-density lipoprotein cholesterol level (mean difference –2.18 mg/dL) [102].

**Table 8:** Effectiveness of digital interventions on other combinations of outcomes, sorted by controls and further sorted by intervention type.

Review		Relevant studies, n (total studies)	Method of synthesis	Interventions	Outcomes	Follow-up	Summary of findings	AMSTAR-2 rating
**Mixed (active and nonactive) controls**								
	Beishuizen et al (2016) [102]	15 (57)	Meta-analysis of RCTs^a^	Internet	Systolic blood pressure, diastolic blood pressure, HbA_1c_^b^ level, cholesterol level, weight, and level of physical activity	3-60 months	Intervention groups had a reduction in systolic blood pressure (mean difference –2.66 mm Hg; 95% CI –3.81 to –1.52), diastolic blood pressure (mean difference –1.26 mm Hg; 95% CI –1.92 to –0.60), HbA_1c_ level (mean difference –0.13%; 95% CI –0.22 to –0.05), and LDL^c^ cholesterol level (mean difference –2.18 mg/dL; 95% CI –3.96 to –0.41). There were larger effects in internet interventions that combined the internet application with human support (blended care).	Low
	Chebli et al (2016) [103]	11 (16)	Narrative synthesis of RCTs	Internet	Cessation and reduction (for both alcohol and smoking)	1-12 months	Internet-based interventions may have a positive effect on smoking cessation. Several studies found that web-based use and number of log-ins were positively associated with quit outcomes. Both studies on alcohol demonstrated positive treatment outcomes in both arms, but there were no differences between the internet-based intervention and control.	Critically low
	Cugelman et al (2011) [105]	30 (30)	Meta-analysis of experimental, quasiexperimental, and correlational studies	Internet	Behavioral outcomes	1 day-7 months	Effect sizes were small but statistically significant (standardized mean difference effect size Cohen *d*=0.19; 95% CI 0.11 to 0.28; *P*<.001; number of interventions, k=30); however, there was a lot of heterogeneity, Cochran's Q test 64.125 (*P*<.001) and I^2^=54.776; the largest effect size was observed when interventions were compared with waitlists and placebos (Cohen *d*=0.28; 95% CI 0.17 to 0.39; *P*<.001; k=18); there was no significant difference compared with sophisticated print interventions (Cohen *d*=–0.11; *P*>.05; k=29).	Critically low
	Webb et al (2010) [113]	85 (85)	Meta-analysis of RCTs	Internet	Smoking abstinence, level of physical activity, alcohol consumption, and dietary behavior	3-12 months	Interventions had a statistically small but significant effect on health-related behavior (Cohen *d*+=0.16; 95% CI 0.09 to 0.23). The effect size of interventions targeting a single area of health behavior was not significantly different to the effect size of those targeting multiple areas of health behaviors, but the numerical difference was in favor of single-area studies (Cohen *d*+=0.17 versus Cohen *d*+=0.12).	Critically low
	Lustria et al (2013) [110]	40 (40)	Meta-analysis of experimental and quasiexperimental studies	Tailored internet-based interventions	Levels of physical activity, fruit and vegetable intake, saturated fat intake, and abstinence from smoking and alcohol consumption	1-24 months	Web‐based, tailored interventions effected significantly greater improvement in health outcomes as compared with control conditions immediately post intervention, Cohen *d*=0.139 (95% CI 0.111 to 0.166; **P*<*.001; k=40). The effect remained at follow‐up, Cohen *d*=0.158 (95% CI 0.124 to 0.192; *P*<.001; k=21), and the correlation between follow‐up time point and effect size was *r*_39_=.004 (*P*=.98, for posttest effects) and *r*_20_=−.176 (*P*=.50, for follow‐up effects), which suggests that length of follow‐up did not significantly influence intervention outcomes. Interventions using tailored websites had a larger weighted mean effect size when compared with nontailored websites (Cohen *d*=0.188) than when they were compared with no‐treatment control conditions (Cohen *d=*0.07; **P*<*.01). There was an extremely small effect of tailored websites frompared to no-treatment control conditions (Cohen *d*=0.08; k=4) and of tailored websites compared with sophisticated print interventions (Cohen *d*=–0.11; *P*>.05; k=29).	Critically low
	Covolo et al (2017) [104]	39 (40)	Narrative synthesis of RCTs	Mobile apps	BMI and waist circumference; various physical activity levels; fruit and vegetable intake, high-sugar food intake, smoking cessation, and number of drinking days	6 months-2 years	Only 25% (10/40) of RCTs found statistical differences between intervention and control groups.	Critically low
	Armanasco et al (2017) [101]	35 (51)	Meta-analysis of RCTs and quasiexperimental studies	Text messaging	Weight, level of physical activity, and smoking cessation	1 to 66 weeks	The overall pooled effect of interventions was Cohen *d*=0.24 (95% CI 0.16 to 0.32; *P*<.001; k=35) using outcome data collected most proximal to the intervention end. 7 studies collected data following a no-intervention maintenance period and showed a small but significant pooled maintenance effect (Cohen *d*=0.17; 95% CI 0.03 to 0.31; *P*=.017; k=7).	Critically low
	Head et al (2013) [107]	12 (19)	Meta-analysis of RCTs	Text messaging	Smoking cessation, weight loss, and level of physical activity	Mean 81.26 days	The overall weighted mean effect size representing the impact of these interventions on health outcomes was Cohen *d*=0.329 (95% CI 0.274 to 0.385; *P*<.001). Correlations between effect size and follow-up (*r*_18_=−.12; *P*=.62) and effect size and retention (*r*_18_=.14; *P*=.56) were not statistically significant. Effect sizes for interventions that employed no-treatment control groups (Cohen *d*=0.369), however, were significantly larger than those that employed alternative comparisons (Cohen *d*=0.226; *Q_B_*=.5.16, df=1; *P*=.02).	Critically low
	De Leon et al (2014) [106]	55 (55)	Narrative synthesis of RCTs	Text messages and emails (prompts)	Smoking cessation, diet intake, and physical activity level	3 weeks-9 months	76% (42/55) of articles found statistically significant positive behavioral outcomes of prompts, the mode by which the prompt was sent did not seem to impact its success.	Critically low
	Rooke et al (2010) [112]	34 (34)	Meta-analysis of RCTs	Computer delivered	Abstinence and reduction for both smoking and alcohol	1-156 weeks	The weighted average effect size (Cohen *d*) was 0.20 (*P*<.001); however, lower effect sizes were associated with studies addressing tobacco use (Cohen *d*=0.14). There was significant heterogeneity between studies targeting tobacco versus alcohol use (*Q*=5.65; *P*=.02), with studies on alcohol consumption producing significantly higher effect sizes than tobacco studies (Cohen *d*=0.26 and 0.12, respectively). Effect sizes were higher for studies in which the comparison condition was an attention/placebo (Cohen *d*=0.22) relative to studies in which the comparison condition was an active comparison (Cohen *d*=0.10); studies employing active treatments as the comparison condition mainly produced effect sizes close to zero.	Critically low
**Nonactive controls**								
	Krebs et al (2010) [109]	76 (88)	Meta-analysis of RCTs	Computer-tailored interventions	Dietary intake of fat, vegetables, fruit; various levels of physical activity, and smoking abstinence over various timescales	1-24 months	The overall effect size was *g*=0.17 (95% CI 0.14 to 0.19) using a fixed effects model and *g*=0.17 using a random effects model (95% CI 0.14 to 0.20). Effects peak from 4 to 12 months postbaseline with a mean effect size of *g*=0.20, and while they decline after 12 months postbaseline, the mean effect size at long-term follow-up (*g*=0.12) remains to be statistically significant (95% CI 0.08 to 0.16). The meta-analysis found a trend for increasing effect sizes across studies that intervened on 1 (*g*=0.15), 2 (*g*=0.21), and 3 (*g*=0.24) areas of behavior, but this trend did not continue with the 1 study that intervened on 4 areas (*g*=0.12).	Low

^a^RCT: randomized controlled trial.

^b^HbA_1c_: glycated hemoglobin.

^c^LDL: low-density lipoprotein.

Of the 3 reviews of mobile interventions, a review of SMS text messaging concluded that they were effective, but the narrative synthesis of apps was the only review in the other combinations category that did not find a preponderance of positive results [104]. The effect sizes for the SMS text messaging interventions were small (Cohen *d*=0.24 [101] and Cohen *d*=0.329 [107]), and the effect sizes in the meta-analysis were heterogeneous [107].

Other modes of intervention continued in the pattern of positive, albeit small, effects. Computer-delivered interventions had a small effect (Cohen *d=*0.20) [112]. Prompts delivered by SMS text messaging or email were effective in changing diet, physical activity, and smoking behaviors [106].

##### Relative Effect of Interventions by Number of Behaviors Targeted

A total of 3 meta-analyses that compared interventions targeting single versus multiple areas of behaviors found that both types of interventions were effective but they differed in whether they found interventions with single or multiple targets to be more effective. A total of 2 meta-analyses of internet-based interventions found that the effect size of interventions targeting a single area of health behavior was not significantly different to the effect size of those targeting multiple areas of health behaviors, but the numerical difference was in favor of single-area studies (Cohen *d*=0.146 vs Cohen *d*=0.121 for multiple-behavior studies [110]; Cohen *d+*=0.17 vs Cohen *d+*=0.12 [113]). However, the computer feedback–based meta-analysis found a trend for increased effect sizes across studies that intervened in 1 (*g*=0.15), 2 (*g*=0.21), and 3 (*g*=0.24) areas of behavior, but this trend did not continue with the one study that intervened in 4 areas (*g*=0.12) [109]. The review covered diet, physical activity, smoking cessation, and mammography.

##### Relative Effect of Intervention by Area of Behaviors Targeted

The review of SMS text messaging interventions found that interventions targeting smoking cessation and physical activity were more successful than interventions targeting other areas of health behavior, including alcohol and weight loss [107]. In contrast, a review of computer-delivered interventions found that interventions targeting smoking had statistically significantly lower effect sizes than those targeting alcohol (Cohen *d*=0.26 and 0.12, respectively) [112].

##### Effect of Other Combination Interventions Compared With Nonactive Controls

Digital interventions had their largest effect sizes when compared with nonactive controls; however, the effect sizes were generally small. Internet interventions had the largest effect size when compared with waitlists and placebos, but the effect sizes were small: Cohen *d*=0.28 [105] and Cohen *d*=0.22 [107]. Tailored websites had extremely small effect sizes when compared with no-treatment control conditions (Cohen *d=*0.07), and the effects were not statistically significant compared with nontailored print materials [110]. There were medium effect sizes for SMS text messaging interventions that employed no-treatment control groups (Cohen *d*=0.369) [107].

##### Effect of Other Combination Interventions Compared With Active Controls

The effects of digital interventions compared with active controls were very small or nonexistent.

A total of 2 meta-analyses found that there was an extremely small effect size (Cohen *d*=0.08) [110] or no significant difference [105] when internet-based interventions were compared with sophisticated print interventions. There were larger effects in internet-based interventions that combined internet application with human support (blended care) [102]. Computer-delivered interventions had very small effect sizes when the comparison condition was an active comparison (Cohen *d*=0.10); studies employing active treatments as the comparison condition mainly produced effect sizes close to zero [112]. SMS text messaging interventions had small effect sizes compared with active comparisons (Cohen *d*=0.226), which was statistically significantly smaller than their effect size compared with the no-treatment control groups [107].

Although digital feedback was effective, it seems that it was no more effective than feedback delivered by nondigital means, and 2 reviews that examined feedback interventions found that the medium did not affect behavior change (SMS text messaging, print, email, telephone, newspaper articles [106] and print, computer, telephone, etc [109]).

##### Sustainability of Effects at Follow-Up

There was mixed evidence on the sustainability of interventions. A meta-analysis of internet-based interventions found the largest effect size at 1 month to 4 months [105], but a meta-analysis of computer-tailored interventions found that effects peaked from 4 months to 12 months postbaseline [109]. A total of 3 meta-analyses found that the correlation between effect size and follow-up was not significant for internet-based [110], SMS text messaging [107], or computer-delivered interventions [112].

The effects did seem to decline in the long run, and the 2 meta-analyses that explicitly examined effectiveness 1 year postintervention (for internet-based and computer-delivered interventions) found that the effect size declined, although it remained statistically significant [102,109].

#### Adherence (Considered for All Behavioral Areas)

Typically, adherence data were not reported. Where reported, there were generally decreases in program usage over the intervention period [75]. Rates of attrition were variable and sometimes very high, for instance, in one diet review, it was 0% to 84% [61] and in 2 smoking reviews it was ≥60% [86] and 35% to 84% (median 70%) [93]. Bucking the trend, a couple of reviews reported high adherence to digital physical activity interventions [46,60]. Many reviews found a dose-response relationship, whereby the effectiveness of the intervention was positively associated with dietary usage [20,21,61], weight loss [47], smoking [87,90,93,103], and alcohol [79]. However, there was no unanimity, for instance, one combination review found that the attrition rate was lower in internet-based interventions than in face-to-face settings [103] and 2 others found no evidence that retention influenced outcomes [107,110].

## Discussion

### Principal Findings

We reviewed 94 systematic reviews and meta-analyses that examined the effectiveness of digital interventions in changing health-related behavior and improving health outcomes in the areas of diet, physical activity, diet and physical activity combined, alcohol consumption, and smoking cessation, alone or in any other combination. The effectiveness of digital interventions differed according to the area of health behavior reviewed. Small positive effects were evident in smoking- and alcohol-related interventions. Similar findings were observed in the combined diet and physical activity interventions, as well as in other outcome combinations. However, there was little evidence of the effectiveness of stand-alone diet interventions, and evidence of the effectiveness of physical activity interventions was mixed, with some consistently positive evidence for mobile interventions and some promising evidence for exergaming. Digital interventions were most effective in the short-to-mid term (approximately 3 to 6 months), but there was insufficient evidence about their long-term effect. Typically, they were more effective than no intervention. There is mixed evidence on their effectiveness compared with nondigital interventions.

Our secondary objective was to identify differences in effectiveness between the modes of delivery of digital interventions. We identified internet-based interventions to be one of the more effective interventions for each area studied, except for physical activity alone. Mobile interventions were particularly effective for diet and physical activity combined (medium effects), but they were also effective for alcohol and smoking (small effects) and physical activity alone. Social media interventions were not effective for diet and physical activity combined (weight loss interventions), they had mixed effects for diet, and there was limited evidence for other areas. Computer-delivered technologies had small effects for smoking and alcohol consumption, but the effects for diet and physical activity were mixed.

The effect sizes reported in the reviews were generally below the National Institute for Health Care Excellence (NICE) guidelines for effectiveness of interventions, although it was often difficult to compare results with NICE guidelines, as different measures were used.

For weight management, NICE guideline PH53 (Recommendation 13) states that commissioned lifestyle weight management programs should have at least a 60% completion rate and should be likely to lead to an average weight loss of at least 3%, with at least 30% of participants losing at least 5% of their weight [114]. It should be noted that this is based on all participants, that is, those who attend at least one session. For those *completing* the service, that is, attending at least 75% of sessions, the key performance indicator of 50% losing at least 5% has been set [115]. In contrast, most reviews reported changes in weight in kilograms or changes in BMI, rather than percentage weight loss. The highest effect sizes for BMI in our review were −0.92 kg/m^2^ [49] and −0.43 kg/m^2^ [38], which are extremely unlikely to represent a 3% or 5% weight loss in individuals with overweight or obesity. The largest changes in weight found in the reviews of digital interventions were −2.71 kg in one study [72] and −2.56 kg in one meta-analysis [71]. In comparison, the Hartman-Boyce evidence review that supports NICE guideline PH53 found an average effect size of −2.59 kg for face-to-face services at 12 months (intention-to-treat analysis) [116]. Most effect sizes from digital interventions did not reach the effect size of face-to-face services.

For smoking, the national outcome measure for stop smoking services is 4-week quits. Smokers attempting to stop without additional support generally have a success rate of 25% at 4 weeks for carbon monoxide–validated quits and a success rate of about 35% at 4 weeks for self-reported quits [117]. Therefore, to show an impact, services must achieve success rates equivalent to or in excess of these rates that smokers achieve without support. Patients who receive stop smoking service support (behavioral support and pharmacotherapy) are 3 times more likely to quit than those with no support [118], and there is a cessation rate of 35% for brief intervention services but only at the 4-week point [117]. It is difficult to compare the results of our review with these services because the outcomes of digital smoking interventions are often expressed as ORs for smoking cessation rather than cessation rates. The only available review that used cessation rates demonstrated a cessation rate of 14.8% [92]. This is lower than the observed rate from stop smoking services or brief advice, but the follow-up point was considerably later and cessation rates decreased over time.

For alcohol, the average weekly reduction in drinking from brief advice interventions is 20 g of alcohol (about 2.5 UK units) [119], so the reductions in weekly alcohol consumption achieved by digital alcohol interventions are comparable when including all interventions (22 g of alcohol or 3 UK units) but lower if one restricts attention to high-quality evidence (11 g of alcohol or 1.5 UK units) [77].

To the extent that the effectiveness of digital interventions is below the NICE guidelines, doctors and organizations should be cautious about recommending them to patients who would benefit from behavior change in the 4 areas of our review. However, it may be valuable to recommend them to people who refuse a face-to-face intervention. On the basis of the evidence that we found, digital interventions for weight loss should combine diet and exercise unless they are mobile interventions targeting physical activity.

For digital interventions for both smoking and combined diet and physical activity, there were studies showing significant health effects but not significant behavioral effects. This seems paradoxical. However, it is possible that even a small increase in physical activity or a small improvement in diet, which may not be statistically significant, can improve health markers over an intervention period, particularly in the most inactive and less fit individuals. Light physical activity is beneficial for health outcomes, including cardiometabolic risk factors [120]. We also noted that, although statistically significant, the health effects are small and possibly not meaningful.

### Limitations

Owing to the rapid nature of our review, we did not perform full hand searches or consult experts. This may mean that we overlooked some reviews. At the other end of the spectrum, by reviewing reviews, there is the possibility that some studies were double counted if they were covered in more than one review.

Our ability to make inferences from the literature reviewed is limited for the following reasons.

Heterogeneity was consistently high across reviews. Heterogeneity of effects probably reflects heterogeneity of interventions, which could be a consequence of rapid advances in digital devices and systems. There were also heterogeneous outcome measures. As the reviews covered different types of digital interventions and outcome measures, it was difficult to make comparisons. Differing outcome measures may have differentially impacted the effectiveness of modes of intervention or the general effectiveness of interventions in the areas we investigated. For instance, smaller effect sizes were reported in studies addressing smoking use than in studies on alcohol consumption, possibly because studies addressing alcohol use tended to use reductions in drinking behavior as their outcome variable, while studies addressing smoking use tended to apply the more stringent standard of abstinence [112].

Follow-up times were relatively short, so we cannot know if behavior change would be sustained in the long term. Some trials only provided behavioral data, so we cannot be sure of health outcomes. A review of physical activity found that the average rate of sustained use of digital health interventions over 10 weeks was 50% [64]. This is consistent with the findings of another systematic review on physical activity apps, which concluded that apps are effective in the short term (up to 3 months) but not longer [121].

Most trials reported intent-to-treat analysis, and typically, adherence data were not reported. This makes it difficult to assess nonsignificant effects to determine whether they resulted from ineffectiveness of treatment or from nonadherence. Where attrition rates were reported, they were often high.

Anecdotally, digital interventions are being used both to supplement and replace face-to-face services. However, most reviews did not discriminate between these functions. In the domain of weight loss, 5 reviews reported enhanced effects on weight loss in interventions that incorporated personal contact or counseling [51,54,57,62,74]. One meta-analysis showed that infrequent in-person treatment was superior in limiting weight gain to computer-based interventions (mean difference 0.5 kg). Digital interventions that particularly benefit from involving people alongside are thought to include sensitive tailoring of feedback [57,62] and social support [54].

Confidence ratings were critically low in 79 of 93 reviews (85%). However, when isolated, those reviews that were rated critically low presented findings that were consistent with the overall findings: equivocal evidence on effectiveness for diet or physical activity outcomes but consistent findings of short-term effects for alcohol, smoking, and other combined outcomes.

Even when the AMSTAR-2 [16] ratings were moderated (so that justifying any publication language inclusion criterion and providing a list of justifications for excluding reviews were no longer considered critical flaws), 74 of 93 (80%) reviews were rated critically low. During the generation of confidence ratings, it was noted that many reviews failed to satisfy items 2 and 13. These are considered critical items for all review types. Item 2 specifies that, as a minimum, reviews state that a protocol containing research questions, search strategy, inclusion/exclusion criteria, and a risk of bias assessment was completed before conducting the review. Item 13 dictates that reviews should account for the risk of bias in individual studies when interpreting/discussing the results of the review. The inclusion of these items in the AMSTAR-2 rating may represent an aspiration to improve standards. Our AMSTAR-2 quality ratings are consistent with other evidence that suggests that it is possible to satisfy PRISMA standards yet still have poor methodological quality [122]. However, judging reviews according to such high standards, such that they are virtually all rated as being very low, masks the differences in quality.

### Future Research Work

This is the first review of reviews on the effectiveness of digital interventions with such a large scope. It summarizes the state of our knowledge of digital interventions for health improvement behaviors in nonclinical populations. However, the literature could be developed to be more helpful for professionals and organizations who need to decide whether to promote digital interventions, which ones to promote, which areas of behaviors to promote them for, and who to promote them to.

For policy purposes, reviews with mixed controls are of limited use. It matters whether a digital intervention is being compared with no intervention or an active nondigital control. It also matters whether the intervention is a stand-alone digital intervention or whether digital is being used as an adjunct to face-to-face services. We cannot assume that a digital intervention that is successful as an adjunct will also be successful as a stand-alone intervention. Therefore, reviews are needed to separately summarize the evidence base for these different ways of using digital interventions. It could be helpful to have well-structured and coordinated reviews that collate a high-level picture for each area of behavior, which can be updated on a regular basis. We need comparisons with national measures of effectiveness, such as the NICE guidelines, to more easily influence policy.

We also need reviews that can help us determine which interventions are most effective. In the future, it would be helpful to conduct comparative research on the mode of delivery of digital interventions (including comparisons of effect sizes), so that we can determine the most promising interventions for further development. There was also a lack of evidence about the long-term effects of interventions, and more studies on the sustainability of behavior change after digital interventions are needed. It would be especially useful to have this information in comparison with active controls.

Professionals also need to know who should be recommended digital interventions. Therefore, it would also be useful to know whether effect sizes are consistent across various subgroups of the population or whether digital interventions have different effects in different subgroups. We were not specifically looking for this information, but the reviews that were surveyed had mixed findings about whether the effectiveness of digital interventions varied with sex and age. Three meta-analyses found no significant association between sex and effect size or age and effect size [102,109,110]. However, one study found that the effect of interventions declined as age increased [105]. There may also be sex-based differences in adherence, and it is plausible, though not proven, that adherence moderates effect size. One narrative synthesis found that women and middle-aged participants were more likely to use web-based intervention services than men and younger participants, and women were more adherent to the overall intervention [103]. There is even less information about differential effects according to socioeconomic status (SES). In the domain of smoking, one review found that the relative effectiveness of technology-based interventions appeared to be comparable between low- and high-SES groups [83].

The acceptability of digital interventions to their target users also warrants further study. In one review of digital interventions of addictive behaviors, participants expressed a preference for internet-based services because of the convenience and increased confidentiality, and individuals who might not otherwise seek treatment said they would consider an internet-based intervention [103].

Providers may be drawn to digital tools in the hope that they are cost-effective. While not the purpose of our review of reviews, we noted insufficient evidence in the reviews to draw any preliminary inferences about the cost-effectiveness of digital interventions. The evidence from the reviews was mixed. There was evidence in favor (internet-based health interventions [41]), evidence against (adaptive e-learning interventions [22]), and mixed evidence: 1 of 3 (33%) web-based interventions was cost-effective compared with in-person interventions at 6 months [52]. Cost-effectiveness may also depend on whether digital interventions supplement or replace face-to-face interventions. Cost-threshold analyses indicated that some form of electronic intervention is likely to be cost-effective when added to nonelectronic behavioral support, but there is substantial uncertainty with regard to determining the most effective (thus most cost-effective) type of electronic intervention, which warrants further research [84]. Future work will need to investigate cost-effectiveness to allocate resources to developing the most promising digital tools.

### Conclusions

Our review of reviews summarizes the state of our knowledge of digital interventions for health improvement behaviors in nonclinical populations. We found positive but small effects for digital interventions that targeted diet and physical activity combined, greater effects—but still small—for smoking and alcohol consumption, and positive, medium-sized effects for mobile interventions for physical activity alone. More high-quality research is needed to assess the sustainability of the effects of digital interventions in the long term, the differences between modes of delivery for digital interventions, their effect on different population subgroups, their cost-effectiveness compared with existing behavior change approaches, and in particular whether they are better used as an adjunct to or replacement for face-to-face treatment. We need the answers to these questions to be able to make an informed decision about whether digital behavior change tools should be integrated into the NHS Health Check program.

## Appendix

Multimedia Appendix 1 PRISMA (Preferred Reporting Items for Systematic Reviews and Meta-Analyses) checklist.

Multimedia Appendix 2 Search strategy.

Multimedia Appendix 3 Data extraction form.

Multimedia Appendix 4 List of excluded reviews.

Multimedia Appendix 5 Breakdown of the reviews included in the study.

Multimedia Appendix 6 Key characteristics of the included studies.

## Abbreviations

CVD: cardiovascular disease
NHS: National Health Service
NICE: National Institute for Health Care Excellence
PHE: Public Health England
PRISMA: Preferred Reporting Items for Systematic Reviews and Meta-Analyses
RCT: randomized controlled trial
RR: risk ratio
SES: socioeconomic status
SMD: standardized mean difference
