# Supplementary material for: Effectiveness of Digital Interventions for Reducing Behavioral Risks of Cardiovascular Disease in Nonclinical Adult Populations: Systematic Review of Reviews
Source: J Med Internet Res. 2021 May 14;23(5):e19688. doi: 10.2196/19688 (PMC8164125; doi:10.2196/19688)
Supplement: Multimedia Appendix 2 [file jmir_v23i5e19688_app2.docx]

**Search strategies used for the review on the effectiveness of digital interventions for diet, physical activity, alcohol and smoking**

**Ovid Medline**

| 1. TELEMEDICINE/ or Therapy, Computer-Assisted/ or User-Computer Interface/ or Software Design/ or MULTIMEDIA/ or Computers, Handheld/ or Videotape Recording/ or Internet/ or Social Networking/ or Blogging/ or Social Media/ or Electronic Mail/ or Cell Phones/ or Text Messaging/ or Smartphone/ or Mobile Applications/ or WEARABLE ELECTRONIC DEVICES/ or Video Games/ or Virtual Reality/ |  |
| --- | --- |
| 2. ((digital* or digitis* or digitiz* or electronic*) adj3 (intervention* or therap* or treatment* or medicine* or medical* or health* or monitoring or clinical* or communicat* or technol* or media* or device* or platform* or forum* or community* or communities* or discussion*)).tw,kw. |  |
| 3. (telemed* or tele-med* or telehealth* or tele-health* or telecar* or tele-car*).tw,kw. |  |
| 4. (ehealth* or e-health* or mhealth* or m-health* or mobile health*).tw,kw. |  |
| 5. ((laptop or palm or handheld or tablet or pda or pc) adj2 comput*).tw,kw. |  |
| 6. ((mobile* or cell* or tablet*) adj (phone* or telephone* or handset* or hand-set*)).tw,kw. |  |
| 7. (smartphone* or smart-phone* or smart telephone* or iphone* or i-phone* or ipad* or i-pad* or blackberry* or smartwatch* or smart-watch* or android or device-based or mobile-based or podcast*).tw,kw. |  |
| 8. ((mobile or electronic* or digital*) adj2 (device* or tablet*)).tw,kw. |  |
| 9. ((mobile or electronic* or digital* or device* or software*) adj3 application*).tw,kw. |  |
| 10. (app or apps or wearable* or online* or on-line* or internet* or www or web or website* or webpage* or portal or search engine*).tw,kw. |  |
| 11. (e-mail* or email* or electronic mail*).tw,kw. |  |
| 12. (text messag* or texting or texter* or texted or SMS or short messag* or multimedia messag* or multi-media messag* or mms or instant messag* or picture messag* or audio messag*).tw,kw. |  |
| 13. (Facebook* or YouTube* or Twitter* or LinkedIn* or Pinterest* or Google* or Tumblr* or Instagram* or WhatsApp* or Reddit* or Flickr* or SnapChat* or Yahoo* or Bing* or MSN* or Wikipedia* or Web 2* or alexa or siri or fitbit*).tw,kw. |  |
| 14. (social media* or social network* or blog* or vlog* or video-blog* or gaming or game or games or gamification or wii fit or discussion board* or online forum*).tw,kw. |  |
| 15. ((virtual or augmented) adj3 reality).tw,kw. |  |
| 16. Speech Recognition Software/ |  |
| 17. ((voice* or speech or speak*) adj3 response* adj3 (interact* or unit*)).tw,kw. |  |
| 18. IVR.tw,kw. |  |
| 19. 1 or 2 or 3 or 4 or 5 or 6 or 7 or 8 or 9 or 10 or 11 or 12 or 13 or 14 or 15 or 16 or 17 or 18 |  |
| 20. exp EXERCISE/ or exp EXERCISE MOVEMENT TECHNIQUES/ or exp SPORTS/ or exp exercise therapy/ or SEDENTARY LIFESTYLE/ or FOOD HABITS/ or FOOD PREFERENCES/ or Nutrition therapy/ or *DIET/ or Body Mass Index/ or Healthy diet/ or SMOKING/ or SMOKING CESSATION/ or "TOBACCO USE CESSATION"/ or exp "TOBACCO USE"/ or "TOBACCO USE DISORDER"/ or SMOKERS/ or Electronic Nicotine Delivery Systems/ or Vaping/ or "TOBACCO USE CESSATION PRODUCTS"/ or exp Pipe smoking/ or exp ALCOHOL-RELATED DISORDERS/ or exp ALCOHOL DRINKING/ or exp Alcoholic Beverages/ or Drinking Behavior/ |  |
| 21. ((physical* or keep* or cardio* or aerobic or fitness or increas* or more or become or becoming or be or encourag*) adj3 (fit* or activ* or train*)).ti. |  |
| 22. exercis*.ti. |  |
| 23. (sedentary adj3 (behavio?r* or lifestyle* or less or time or change* or changing or modification or modify or modifying or program* or intervention*)).tw,kw. |  |
| 24. diet*.ti. |  |
| 25. ((health* or unhealthy or poor* or chang* or behav* or advic* or recommend*) adj3 (eat* or diet* or food* or nutrition* or weight* or overweight)).tw,kw. |  |
| 26. ((fruit* or vegetable*) adj2 (intake* or consum* or eat* or ate)).tw,kw. |  |
| 27. (ecig* or e-cig* or e-voke* or juul* or vape* or vaping*).tw,kw. |  |
| 28. (waterpipe* or water pipe* or dokha or dokhas or hookah or hookahs or hooka or hookas or shisha or shishas or sheesha or sheeshas).tw,kw. |  |
| 29. (smoking* or smoker* or antismok* or anti smok* or anti-smok*).tw,kw. |  |
| 30. (tobacco* or nicotin* or cigar* or cigs).tw,kw. |  |
| 31. ((Alcohol* or Drunk* or Drink* or beer* or wine* or liqor* or liquor* or spirit* or alcopop* or cider*) adj4 (consum* or misus* or abus* or intoxicat* or inebriat* or excess* or bing* or hazardous or harmful or heavy or heaviest or problem* or risk* or frequen* or behavio?r* or temperance or abstinence or abstain* or stop or stopping)).tw,kw. |  |
| 32. 20 or 21 or 22 or 23 or 24 or 25 or 26 or 27 or 28 or 29 or 30 or 31 |  |
| 33. 19 and 32 |  |
| 38. Health Behavior/ or Health Knowledge, Attitudes, Practice/ or Risk Reduction Behavior/ or Behavior Therapy/ or PSYCHOTHERAPY/ or Cognitive Therapy/ or MOTIVATION/ or Patient Education as Topic/ or Patient acceptance of healthcare/ or Health promotion/ or "Outcome and Process Assessment (Health Care)"/ |  |
| 39. ((behavio?r* or lifestyle* or "life style*") and (change* or changing or modification or modify or modifying or therapy or therapies or program* or intervention* or technique* or establish* or individual*)).ti. |  |
| 40. ((behavio?r* or lifestyle* or "life style*") adj2 (change* or changing or modification or modify or modifying or therapy or therapies or program* or intervention* or technique* or establish* or individual*)).tw,kw. |  |
| 41. motivat*.ti. |  |
| 42. 38 or 39 or 40 or 41 |  |
| 43. 33 and 42 |  |
| 44. limit 43 to yr="2009 - 2019"  45. limit 44 to English language |  |
|  |  |
| 46. limit 45 to "reviews (maximizes specificity)" |  |

The Ovid Embase and Ovid Psycinfo strategies are basically the same and so are not listed.

**Wiley Cochrane Library**

#1 [mh TELEMEDICINE] or [mh “Therapy, Computer-Assisted”] or [mh “User-Computer Interface”] or [mh “Software Design”] or [mh MULTIMEDIA] or [mh “Computers, Handheld”] or [mh “Videotape Recording”] or [mh Internet] or [mh “Social Networking”] or [mh Blogging] or [mh “Social Media”] or [mh “Electronic Mail”] or [mh “Cell Phones”] or [mh “Text Messaging”] or [mh Smartphone] or [mh “Mobile Applications”] or [mh “WEARABLE ELECTRONIC DEVICES”] or [mh “Video Games”] or [mh “Virtual Reality”]

#2 (digital* or digitis* or digitiz* or electronic*) NEAR/3 (intervention* or therap* or treatment* or medicine* or medical* or health* or monitoring or clinical* or communicat* or technol* or media* or device* or platform* or forum* or community* or communities* or discussion*):ti,ab

#3 (telemed* or tele-med* or telehealth* or tele-health* or telecar* or tele-car*):ti,ab

#4 (ehealth* or e-health* or mhealth* or m-health* or "mobile health"):ti,ab

#5 ((laptop or palm or handheld or tablet or pda or pc) NEAR/2 comput*):ti,ab

#6 ((mobile* or cell* or tablet*) NEAR (phone* or telephone* or handset* or hand-set*)):ti,ab

#7 (smartphone* or smart-phone* or "smart telephone*" or iphone* or i-phone* or ipad* or i-pad* or blackberry* or smartwatch* or smart-watch* or android or device-based or mobile-based or podcast*):ti,ab

#8 ((mobile or electronic* or digital*) NEAR/2 (device* or tablet*)):ti,ab

#9 ((mobile or electronic* or digital* or device* or software*) NEAR/3 application*):ti,ab

#10 (app or apps or wearable* or online* or on-line* or internet* or www or web or website* or webpage* or portal or "search engine*"):ti,ab

#11 (e-mail* or email* or "electronic mail*"):ti,ab

#12 ("text messag*" or texting or texter* or texted or SMS or "short messag*" or "multimedia messag*" or "multi-media messag*" or mms or "instant messag*" or "picture messag*" or "audio messag*"):ti,ab

#13 (Facebook* or YouTube* or Twitter* or LinkedIn* or Pinterest* or Google* or Tumblr* or Instagram* or WhatsApp* or Reddit* or Flickr* or SnapChat* or Yahoo* or Bing* or MSN* or Wikipedia* or Web 2* or alexa or siri or fitbit*):ti,ab

#14 ("social media*" or "social network*" or blog* or vlog* or video-blog* or gaming or game or games or gamification or "wii fit" or "discussion board*" or "online forum*"):ti,ab

#15 ((virtual or augmented) NEAR/3 reality):ti,ab

#16 [mh "Speech Recognition Software"]

#17 ((voice* or speech or speak*) NEAR/3 response* NEAR/3 (interact* or unit*)):ti,ab

#18 IVR:ti,ab

#19 {OR #1-#18}

#20 [mh EXERCISE] or [mh “EXERCISE MOVEMENT TECHNIQUES”] or [mh SPORTS] or [mh “exercise therapy”] or [mh “SEDENTARY LIFESTYLE”] or [mh “FOOD HABITS”] or [mh “FOOD PREFERENCES”] or [mh “Nutrition therapy”] or [mh DIET] or [mh “Body Mass Index”] or [mh “Healthy diet”] or [mh SMOKING] or [mh “SMOKING CESSATION”] or [mh "TOBACCO USE CESSATION"] or [mh "TOBACCO USE"] or [mh "TOBACCO USE DISORDER"] or [mh SMOKERS] or [mh “Electronic Nicotine Delivery Systems”] or [mh Vaping] or [mh "TOBACCO USE CESSATION PRODUCTS"] or [mh “Pipe smoking”] or [mh “ALCOHOL-RELATED DISORDERS”] or [mh “ALCOHOL DRINKING”] or [mh “Alcoholic Beverages”] or [mh “Drinking Behavior”]

#21 ((physical* or keep* or cardio* or aerobic or fitness or increas* or more or become or becoming or be or encourag*) NEAR/3 (fit* or activ* or train*)):ti

#22 exercis*:ti

#23 ((behavior* or behaviour* or lifestyle* or less or time or change* or changing or modification or modify or modifying or program* or intervention*) NEAR/3 sedentary):ti,ab

#24 diet*:ti

#25 ((health* or unhealthy or poor* or chang* or behav* or advic* or recommend*) NEAR/3 (eat* or diet* or food* or nutrition* or weight* or overweight)):ti,ab

#26 ((fruit* or vegetable*) NEAR/2 (intake* or consum* or eat* or ate)):ti,ab

#27 (ecig* or e-cig* or e-voke* or juul* or vape* or vaping*):ti,ab

#28 (waterpipe* or "water pipe*" or dokha or dokhas or hookah or hookahs or hooka or hookas or shisha or shishas or sheesha or sheeshas):ti,ab

#29 (smoking* or smoker* or antismok* or "anti smok*" or anti-smok*):ti,ab

#30 (tobacco* or nicotin* or cigar* or cigs):ti,ab

#31 ((Alcohol* or Drunk* or Drink* or beer* or wine* or liqor* or liquor* or spirit* or alcopop* or cider*) NEAR/4 (consum* or misus* or abus* or intoxicat* or inebriat* or excess* or bing* or hazardous or harmful or heavy or heaviest or problem* or risk* or frequen* or behavior* or behaviour or temperance or abstinence or abstain* or stop or stopping)):ti,ab

#32 {OR #20-#31}

#33 [mh “Health Behavior”] or [mh “Health Knowledge, Attitudes, Practice”] or [mh “Risk Reduction Behavior”] or [mh “Behavior Therapy”] or [mh PSYCHOTHERAPY] or [mh “Cognitive Therapy”] or [mh MOTIVATION] or [mh “Patient Education as Topic”] or [mh “Patient acceptance of healthcare”] or [mh “Health promotion”] or [mh "Outcome and Process Assessment (Health Care)"]

#34 ((behavior* or behaviour* or lifestyle* or "life style*") and (change* or changing or modification or modify or modifying or therapy or therapies or program* or intervention* or technique* or establish* or individual*)):ti

#35 ((behavior* or behaviour* or lifestyle* or "life style*") NEAR/2 (change* or changing or modification or modify or modifying or therapy or therapies or program* or intervention* or technique* or establish* or individual*)):ti,ab

#36 motivat*:ti

#37 {OR #33-#36}

#38 #19 and #32 and #37
