# Supplementary material for: Effectiveness of Digital Interventions for Reducing Behavioral Risks of Cardiovascular Disease in Nonclinical Adult Populations: Systematic Review of Reviews
Source: J Med Internet Res. 2021 May 14;23(5):e19688. doi: 10.2196/19688 (PMC8164125; doi:10.2196/19688)
Supplement: Multimedia Appendix 3 [file jmir_v23i5e19688_app3.doc]

# Data collection form

| **Study ID** *(surname of first author and year first full report of study was published e.g. Smith 2001)* |
| --- |
|  |

## General Information

| 1. Date form completed *(dd/mm/yyyy)* | |  |
| --- | --- | --- |
| 1. Name of person extracting data | |  |
| 1. Report title | |  |
| 1. Notes: |  | |

## Eligibility

| **Study Characteristics** | | | **Review Inclusion Criteria** | **Yes/ No / Unclear** | **Location in text**  *(pg & ¶/fig/table)* |
| --- | --- | --- | --- | --- | --- |
| 1. Type of study | | | Systematic review |  |  |
|  |  |  | Meta-analysis |  |  |
|  |  |  | Report |  |  |
|  |  |  | Other analytical design (specify): |  |  |
| 1. Population | | | Adults (not exclusively: pregnant or students) |  |  |
|  |  |  | Non-patient |  |  |
|  |  |  | High-income country |  |  |
| 1. Target behaviour | | | Diet |  |  |
|  |  |  | Physical activity |  |  |
|  |  |  | Smoking |  |  |
|  |  |  | Alcohol |  |  |
|  |  |  | Multiple behaviours  Please specify: |  |  |
| 1. Digital intervention | | | |  |  |
| 1. Types of outcome measures | | | Behaviour |  |  |
|  |  |  | Health |  |  |
| 1. Decision: | |  | | | |
| 1. Reason for exclusion | | |  | | |
| 1. Notes: |  | | | | |
|  | EXCLUDE IF PUBLICATION CONTAINS NO ANALYSIS, IF ANY ANSWER TO (6) IS NO, IF ALL ANSWERS TO (7) ARE NO, IF ANSWER TO (8) IS NO OR IF ALL ANSWERS TO (9) ARE NO. | | | | |

## Study details

|  | | **Descriptions as stated in report/paper** | | **Location in text**  *(pg & ¶/fig/table)* |
| --- | --- | --- | --- | --- |
| 1. Objective | |  | |  |
| 1. Population description (include age, sex, socioeconomic status and ethnicity if available) & numbers | |  | |  |
| 1. Inclusion criteria | |  | |  |
| 1. Exclusion criteria | |  | |  |
| 1. Start date | |  | |  |
| 1. End date | |  | |  |
| 1. Number of studies included | |  | |  |
| 1. Types of studies included | | RCTs |  |  |
|  |  | Quasi-RCTs |  |  |
|  |  | Quasi-experimental studies |  |  |
|  |  | Other | Please specify |  |
| 1. Intervention types (mode of delivery) | | Mobile messaging |  |  |
|  |  | Mobile app |  |  |
|  |  | Website |  |  |
|  |  | Social media |  |  |
|  |  | Videotape recording |  |  |
|  |  | Video games/virtual reality |  |  |
|  |  | Remote communication e.g. online coaching and networks |  |  |
|  |  | Wearable technology |  |  |
|  |  | Other(s) | Please specify: |  |
| 1. Length of follow-up | |  | |  |
| 1. Countries of origin of included studies | |  | |  |
| 1. Quality assessed(yes/no) | | If yes, specify tool used: | |  |
| 1. Method of analysis | | Narrative synthesis |  |  |
|  |  | Meta-analysis |  |  |
|  |  | Other | Please specify: |  |
| 1. Results/findings (include significance/direction where reported) | |  | |  |
| 1. Notes: |  | | | |
| 1. Control/ comparison |  | | | |
