# Supplementary material for: Effectiveness of Digital Interventions for Reducing Behavioral Risks of Cardiovascular Disease in Nonclinical Adult Populations: Systematic Review of Reviews
Source: J Med Internet Res. 2021 May 14;23(5):e19688. doi: 10.2196/19688 (PMC8164125; doi:10.2196/19688)
Supplement: Multimedia Appendix 5 [file jmir_v23i5e19688_app5.docx]

**Table S1.** Breakdown of the study characteristics.

| Target outcome | Number of reviews | | | | | Mode of intervention (some papers cover multiple modes, so numbers may sum to more than the total number of papers) | | | | Controls | | | |
| --- | --- | --- | --- | --- | --- | --- | --- | --- | --- | --- | --- | --- | --- |
|  | Total | Focus only on target outcome | | Extractable data about target outcome | | Internet | Mobile | Social media | Other | Mixed controls (active and nonactive) | Nonactive control (no intervention or minimal control) | Active control |  |
|  |  | Total | Meta-analysis | Total | Meta-analysis |  |  |  |  |  |  |  |  |
|  | | | | | | | | | | | | | |
| Diet behaviors | 20 | 4 [20-23] | 1 [22] | 16 reviews, of which 11 on diet and physical activity (weight loss) interventions [53,56,60,61,63,65,73-75,108] and 5 other combinations [63,73,109,110,113] | 9 reviews, of which 5 on diet and physical activity interventions [60,65,74,75,111] and 4 on other behavioral outcome combinations [73,109,110,113] | 6 reviews, of which 5 on internet-only interventions [63,100,108,110,113] and 1 on internet and mobile intervention, but possible to extract information on the internet alone [99] | 6 reviews, of which 3 covered general mobile phone interventions [23,60,111], 2 focused specifically on apps [21,104], and 1 review of internet and mobile interventions allowed us to extract information on mobile interventions alone [99] | 3 [49,56,65,75] | 6 reviews, of which 2 on interactive computer learning interventions [22,74], 1 on personal digital assistants [20], 1 on computer-tailored interventions [109], 1 on interactive voice response [73], 1 on various interventions [53] | 16 [21-23,53,56,60,63,65,74,99,100,104,108,110,111,113] | 2 [73,109] | 2 [20,104] |  |
| Physical activity | 45 | 23 [24-46] | 6 [29,30,34,35,38,45] | 22 [47,49,53,56,60,61,63,65,68,73-76,99,100,102,104,108-111,113] | 12 [49,60,63,65,73-75,102,109-111,113] | 12 [26,29,33,35,36,47,63,100,102,108,110,113] | 13 reviews [25,27,30,31,38,41,42,44,60,61,68,104,111], of which 1 focused on text messaging [27] and 2 focused specifically on apps [38,104] | 5 [49,56,65,75,76] | 14 reviews, of which 3 on active gaming [37,43,46], 2 on wearables [28,32], 2 on computer-delivered interventions [74,109], 1 on interactive voice response [73], and 7 covering a variety of interventions [24,34,39,40,45,53,99] | 32 [26,27,29,31,33-35,37,39,40-43,45- 47,53,56,60,61,63,65,68,74- 76,99,100,108,110,111,113] | 8 [24,25,30,32,36,49,73,109] | 5 [28,38,44,102,104] |  |
| Diet and physical activity (weight loss) | 35 | N/A^a^ | N/A | 35 [47-62,64-72,74-76,99,100,102,104,107,108,111] | 14 [49,52,58-60,65,66,69-72,74,75,102] | 14 [47,52,57,58,62,69,70,100,102,108] | 8 [50,59,60,68,71,104,107,111] | 6 [49,56,61,65,75,76] | 7 reviews, of which 1 on computer-based [74] and 6 various interventions [48,53-55,66,72] | 31 [47, 48,50,51,53- 57,60- 62,64- 71,74- 76,99,100,102,104,108,111 | 2 [49,72] | 2 [52,58] |  |
| Smoking | 28 | 16 [83-98] | 11 [83-85,88,91,94-98] | 12 [99,100,104,108-111,113] | 7 [99,108-111,113] | 11 [85,87,88,90,91,95,97,100,103,110,113] | 8 reviews on mobile interventions, of which 4 explicitly on SMS text messaging interventions [94,96,107,111], 1 predominantly (10 out of 12 papers) on SMS text messaging [98], 2 further reviews had extractable data on mobile interventions [89,99], and 1 on mobile apps [104] | 2 [56,93] | 8 reviews, of which 1 on web-based computer program intervention [108], 1 on a computer intervention [112], 1 on a computer-tailored intervention (which is not necessarily delivered by computer) [109], 1 on an internet-based or computer intervention [92], 1 on an internet-based and mobile phone intervention [89], and 3 on various interventions [83,84,86] | 25 [56,83,85-100,103,104,107-113] | 2 [84,109] | 1 [104] |  |
| Alcohol | 13 | 6 [77-82] | 4 [77,79,80, 82] | 7 [73,99,103,104,111-113] | 3 [73,112,113] | 6 reviews, of which 5 on internet-alone interventions [78-80,103,113] and 1 on an internet in combination intervention [99] | 2 [111,104] | 0 | 6 reviews, of which 3 on computer-delivered interventions [81,82,112], 1 on computer-delivered and mobile interventions [77], 1 on interactive voice response interventions [73], and 1 on various interventions [99] | 8 [77,78,81,99,103,111-113] | 5 [73,79,80,82,104] | N/A |  |
| Combination | 11 [37,102-107,109,110,112,113] | N/A | N/A | 11 [37,102-107,109,110,112,113] | 8 [37,102,105,107,109,110,112,113] | 5 reviews [102,103,105,113], of which 1 covered online tailored interventions [110] | 3 reviews, of which 1 on mobile apps [104] and 2 on SMS text messaging interventions [101,107] | 0 | 3 reviews, of which 1 on computer-delivered interventions [112], 1 on computer-tailored interventions [109], and 1 on prompts (SMS text messaging and email) [106] | 10 [101-107,110,112,113] | 1 [109] | N/A |  |

^a^N/A: not applicable.
